# Supplementary material for: Neural nano-optics for high-quality thin lens imaging
Source: Nat Commun. 2021 Nov 29;12:6493. doi: 10.1038/s41467-021-26443-0 (PMC8630181; doi:10.1038/s41467-021-26443-0)
Supplement: Supplementary file 1 — Supplementary Information [file 41467_2021_26443_MOESM1_ESM.pdf]

Neural Nano-Optics for High-quality Thin Lens Imaging Supplementary Information

Ethan Tseng[1*], Shane Colburn[2*], James Whitehead[2], Luocheng Huang[2], Seung-Hwan Baek[1], Arka Majumdar[2,3], Felix Heide[1†]

[1] *Princeton University, Department of Computer Science*
[2] *University of Washington, Department of Electrical and Computer Engineering*
[3] *University of Washington, Department of Physics*
[*] *These authors contributed equally to this work*
[†] *Corresponding author. E-mail: fheide@princeton.edu*

Supplementary Note 1: Metasurface Specification

We describe our metasurface specifications in comparison to existing designs in Supplementary Table 1. The proposed metasurface imager demonstrates a large aperture size of $500\,\mu m$, which is more than four times larger in area than other works, while maintaining a low f-number of 2. Our meta-optic is polarization insensitive and enables imaging across the full visible spectrum of $400\,nm$ to $700\,nm$. We evaluate this operating bandwidth by computing the PSF's correlation coefficient (Supplementary Fig. 1) with wavelength and defining the bandwidth as the range across which the coefficient remains above 0.5. This metric informs us how similar the PSF is as a function of wavelength, which is critical given that the PSF can only be experimentally calibrated at discrete wavelengths even if broadband light is captured by the meta-optic while imaging. A higher correlation coefficient implies a greater similarity and facilitates robust reconstruction. The wavelength range over which the coefficient exceeds 0.5 thus defines the operating bandwidth. This coefficient is computed as a normalized inner product of the PSF with itself at a fixed reference wavelength. In this analysis, we set the reference wavelength to $511\,nm$.

Supplementary Table 1: Metasurface and imaging specifications of the proposed approach and existing methods. The proposed neural nano-optics and deconvolution method are learned end-to-end using first-order stochastic optimization and image quality losses. In contrast, prior works relied on traditional hand-crafted design metrics. The physical nano-optic demonstrates a significant increase in aperture size and FOV while maintaining low f-number, operates within the visible spectrum, and is insensitive to the polarization state of the light field. Our large aperture was designed by jointly optimizing *an order of magnitude more nano-scatterers* while remaining computationally efficient as the entire learning process takes a total of 18 hours.

Method	Aperture (μm)	f/#	# of Nano-Scatterers	Pitch (nm)	FOV	Bandwidth (nm)	Polarization	Design Strategy	Notes
Neural Nano-Optics	500	2	1.6×10^6	350	40°	400 – 700	Insensitive	Neural Design	
Ndao et al.[1]	20	7.5	2.3×10^3	370	8°	640 – 1200	Insensitive	Phase Slope and Intercept Method	1
Chen et al.[2]	26.4	2.54	3.4×10^3	400	22°	460 – 700	Insensitive	Dispersion engineering	1
Colburn et al.[3]	200	1	1.6×10^5	443	20°	400 – 700	Insensitive	Computational imaging	2
Chen et al.[4]	220	25	2.4×10^5	400	30°	470 – 670	Sensitive	Dispersion engineering	3
Wang et al.[5]	50	4.7	5.2×10^4	120	12°	400 – 660	Sensitive	Dispersion engineering	1,4
Shrestha et al.[6]	200	4	N/A	N/A	14°	1200 – 1650	Insensitive	Dispersion engineering	1,5
Khorasaninejad et al.[7]	200	2.425	1.3×10^5	480	23°	490 – 550	Sensitive	Dispersion engineering	1
Wang et al.[8]	55.55	1.8	8.0×10^3	550	31°	1200 – 1680	Sensitive	Dispersion engineering	1
Arbabi et al.[9]	240	3.54	8.3×10^4	740	16°	1450 – 1590	Sensitive	Dispersion engineering	1

[1] FOV was not reported so we estimated FOV assuming aperture diameter equals sensor size.
[2] FOV is determined from off-axis simulations which were taken up to $\pm 10°$.
[3] FOV is determined for a Strehl ratio of 0.8.
[4] In this design, the reported pitch corresponds to the side length in a hexagonal lattice.
[5] Reported for the M2 design, which has the largest aperture among the designs proposed in this work.

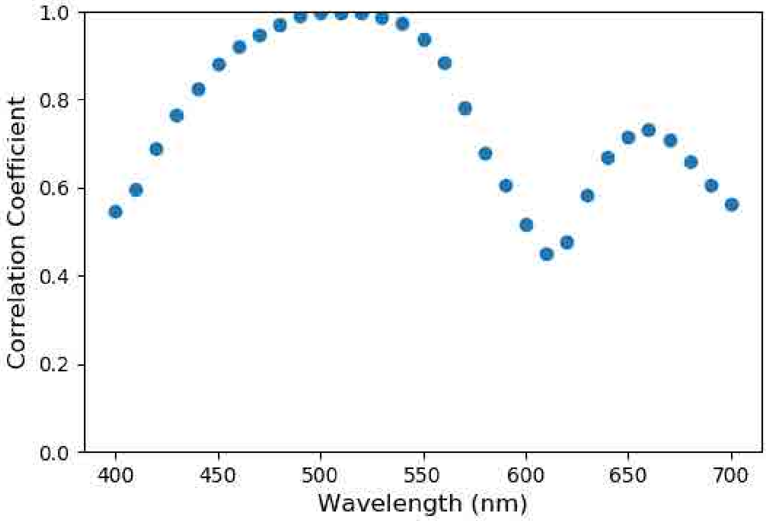

Supplementary Figure 1: Simulated PSF correlation coefficient for our neural nano-optic, where the range over which the coefficient exceeds 0.5 determines the operating bandwidth. Our design supports imaging over the visible spectrum from 400 nm to 700 nm.

Supplementary Note 2: Evaluating Design Capability

In achromatic metasurface design, there are several competing metrics that when individually optimized will come at the expense of the others. For dispersion-engineered meta-optics, for example, one can achieve low f-number but only by reducing the operating bandwidth, having a smaller aperture diameter, or by doing both of these. Reducing aperture size, however, limits light collection, whereas a narrower operating bandwidth would cause strong chromatic aberrations. If the f-number is instead increased, it relaxes the constraints on bandwidth and diameter but at the cost of image resolution. Currently, however, there is no clear metric for simultaneously evaluating all of these competing performance requirements and as such we define a custom quantity, which we term the Diffractive Lens Achromatic Capacity (DLAC), which is the product of the Fresnel Number and Fractional Bandwidth. Maximizing the Fresnel Number, which for paraxial systems is proportional to the NA-aperture product, captures the notion of how maintaining both a low f-number and large aperture diameter is challenging. The fractional bandwidth, the wavelength range divided by center wavelength, evaluates a normalized version of the bandwidth for equal comparison of different designs. For achromatic meta-optics, maximizing either Fresnel Number or fractional bandwidth comes at the expense of the other and by combining these into a single quantity, we can better evaluate the overall capability of a design. A large DLAC is only achievable by simultaneously maintaining low f-number, a large aperture relative to the center wavelength, and a wide bandwidth relative to the center wavelength. Even without considering the computational reconstruction, we achieve a DLAC of 247.9, outperforming all previously demonstrated metasurface imaging systems. Note that DLAC does not include the computational reconstruction, which we measure at the output of the entire imaging pipeline in the remainder of this document.

Supplementary Table 2: Comparison of existing achromatic metasurface designs using our Diffractive Lens Achromatic Capacity (DLAC) metric. The DLAC is a dimensionless parameter given by the product of Fresnel Number and Fractional Bandwidth. Even though this metric does not consider the quality of the computational reconstruction, our neural nano-optic design already outperforms all previously demonstrated works.

Method	DLAC	Design Strategy	Notes
Neural Nano-Optics	247.9	Neural Design	
Ndao et al.[1]	1.8	Phase Slope and Intercept Method	
Chen et al.[2]	7.4	Dispersion engineering	
Colburn et al.[3]	198.3	Computational imaging	
Chen et al.[4]	5.4	Dispersion engineering	
Wang et al.[5]	9.8	Dispersion engineering	
Shrestha et al.[6]	11.1	Dispersion engineering	[1]
Khorasaninejad et al.[7]	18.3	Dispersion engineering	
Wang et al.[8]	7.1	Dispersion engineering	
Arbabi et al.[9]	4.1	Dispersion engineering	

[1]Reported for the M2 design, which has the largest aperture among the designs proposed in this work.

Supplementary Note 3: Enabling Commodity Large Aperture Meta-Optics

High-throughput Fabrication for Commodifying Meta-Optics The meta-optics in the visible wavelength range comprise sub-micron feature scatterers that are difficult to reliably fabricate in a high-throughput manner. Most existing metasurfaces are fabricated via electron-beam lithography, which is low throughput and cannot be used for large numbers of metasurfaces – the cost and time for fabrication grows rapidly as the pattern area increases. Photolithography could be used in place of electron-beam tools, but most traditional photolithography techniques (e.g., contact lithography or laser direct writing) have a resolution limit exceeding 1 μm. Furthermore, most dispersion engineering metasurfaces rely on scatterers comprising multiple, coupled nano-antennas per sub-wavelength unit cell, requiring features even smaller than those of conventional meta-optics, complicating manufacturing and limiting it to only the most high-cost and state-of-the-art manufacturing equipment that is not nearly as widely available. Rather than relying on such scatterer designs and traditional photolithography systems, mass manufacturing will be achievable by leveraging established, high-throughput processes as well as by exploiting our end-to-end design framework with relaxed feature sizes compared to those often found in dispersion engineering designs. While our scatterers consist of a single nanopost with feature size within 100 nm to 290 nm, dispersion engineering approaches often exploit multiple nanostructures per unit cell, entailing smaller feature sizes which frequently go down to 50 nm or less to still satisfy the sub-wavelength pitch characteristic of a metasurface. Instead of using electron-beam lithography, deep ultraviolet (DUV) or immersion lithography systems can achieve feature sizes of $\sim$100 nm but can simultaneously accommodate manufacturing of large numbers of metasurfaces across batches of wafers. These processes are standard in integrated circuits (IC) manufacturing and commodification of meta-optics could leverage these well-developed techniques.

Achieving Larger Aperture Metasurface Fabrication Although we used a relatively large 500 μm aperture in this work, at four times the area of the largest previous full-color metasurface imager, designing even larger aperture sizes will allow for greater light collection and could enable decimeter-scale or meter-scale optics for telescopes. However, even commercial DUV and immersion lithography systems have a limit on die size, which is too small for a single mask to accommodate a decimeter-scale metasurface aperture. Step-and-repeat lithography is the workhorse for patterning of sub-micron features in the IC industry. A mask pattern is projected via optical reduction onto a wafer substrate. The stepper repeats this exposure many times, creating many identically patterned chips on a single substrate. After dicing, each chip is a self-contained device whose size ranges from a few millimeters to more than a centimeter. Instead of conventional step-and-repeat, one could apply a "step-and-stitch" technique capable of creating continuous patterns consisting of multiple individual exposures. This technique would require specially designed masks that seamlessly match up along the borders with their respective neighbors. Stitching is possible due to the extremely precise positioning capability (single-digit nanometer range) of modern lithography steppers. Lastly, even though our forward model is three orders of magnitude more memory efficient than FDTD, efforts to design even larger optics were hampered by limited GPU memory, which can be mitigated by exploiting symmetry in the metasurface mask in the future.

Supplementary Note 4: Differentiable Proxy-Based Metasurface Image Formation

Metasurface Phase Determination and Point Spread Function Calculation The proposed differentiable metasurface model approximates the optical response of the scatterers and then leverages Fourier optics for memory-efficient light propagation (Supplementary Fig. 3). To design the nanoposts, we calculated transmission coefficients using rigorous coupled-wave analysis (RCWA). Our design consists of square cross section, silicon nitride ($n = 2.0$) nanoposts with a thickness of $705\,\text{nm}$ and a pitch of $350\,\text{nm}$ atop a fused silica ($n = 1.5$) substrate also with thickness $705\,\text{nm}$ in simulation. As discussed in the main manuscript, to ensure a differentiable mapping from phase to duty cycle, the phase as a function of duty cycle must be injective. To ensure this, we employ an effective index approximation of the unit cell. While this calculates an approximate transmission coefficient by only considering the DC component of the permittivity to ensure differentiability with respect to the proxy phase, the transmission coefficient of our design does not change appreciably upon including higher Fourier orders with the exception of a few resonances (Supplementary Fig. 2). The simulated full metasurfaces of the main manuscript were modeled by means of the band-limited angular spectrum method where we map the RCWA-computed transmission coefficient to each pixel.

To enable differentiation with respect to duty cycle, we fit this phase data to a polynomial proxy function of the form

$$d(r) = \sum_{i=0}^{N} b_i \left(\frac{\phi(r)}{2\pi} \right)^{2i}, \tag{1}$$

where $d(r)$ is the required duty cycle at a position r from the optical axis on the metasurface, $\phi(r)$ is the desired phase at that same position for the nominal wavelength λ_0, and the parameters b_i are fitted. We set $\lambda_0 = 452\,\text{nm}$ for all of our experiments. Note that $\phi(r)$ is the same as defined in Eq. 1 of the main manuscript. For our square nanoposts we only require expansion up to $N = 2$. After applying this inverse mapping to determine the required physical structure, we compute the phase for the other wavelengths by means of a second proxy function that maps a combination of the nanopost duty cycle and incident wavelength to an imparted phase delay. We model this again by fitting the pre-computed transmission coefficient of our scatterers under an effective index approximation but this time with a polynomial of the form

$$\widetilde{\phi}(r, \lambda) = \sum_{n=0}^{N} \sum_{m=0}^{M} c_{nm} d(r)^n \lambda^m, \tag{2}$$

where λ is a non-nominal wavelength and N and M are the number of orders in duty cycle and wavelength respectively. In the design used for this work, the only non-zero coefficients correspond to terms where the combined degree of d and ϕ is 2 or lower.

Essential in accurately modeling our metasurface is the quality of these fits. Using linear least squares, we find that the determined polynomial fits well to the underlying transmission coefficient

data $\mathcal{C}_{\text{META}}$, as is indicated by their agreement shown in Supplementary Fig. 3. The fitted polynomial coefficients for the inverse and forward mappings are presented in Supplementary Tables 3 and 4, respectively. The computed R-squared coefficients for the inverse and forward mappings were 0.9994 and 0.9998, respectively.

The fixed parameters $\mathcal{C}_{\text{META}}$ determine our metasurface proxy function f_{META} which operates on the optimizable parameters $\mathcal{P}_{\text{META}} = \{a_i | i = 1, \ldots, n\}$, which as described in the main manuscript are the n coefficients of the metasurface phase function. Thus, given an input field angle θ our f_{META} computes a spatially variant PSF as

$$\text{PSF}_\theta = f_{\text{META}}(\theta, \mathcal{P}_{\text{META}}, \mathcal{C}_{\text{META}}). \tag{3}$$

Differentiable Sensor Noise We model the sensor noise as a per-pixel Gaussian-Poisson noise. Specifically, if $x \in [0, 1]$ is the input signal at some sensor pixel location and $f_{\text{SENSOR}}(x)$ is the noisy measurement, then

$$f_{\text{SENSOR}}(x) = \eta_g(x, \sigma_g) + \eta_p(x, a_p), \tag{4}$$

where $\eta_g(x, \sigma_g) \sim \mathcal{N}(x, \sigma_g^2)$ is the Gaussian noise component and $\eta_p(x, a_p) \sim \mathcal{P}(x/a_p)$ is the Poisson noise component. Thus, our f_{SENSOR} function is a-priori determined by the noise parameters $\mathcal{C}_{\text{SENSOR}} = \{\sigma_g, a_p\}$. We use the calibration method described in Foi et al.[10] to estimate these parameters. We determined $\sigma_g = 1 \times 10^{-5}$ and $a_p = 4 \times 10^{-5}$ and we use these values for all of our optimization experiments and synthetic evaluations.

In order to employ auto-differentiation for our end-to-end optimization pipeline, we require that gradients can flow from the endpoint loss all the way back to the meta-optic. For end-to-end differentiability we thus implement every step of our image formation and deconvolution in a differentiable manner. This includes the sensor noise as well, so we utilized several techniques for incorporating differentiability into these stochastic functions. Specifically, differentiable Gaussian noise is implemented using the Reparameterization trick[11]:

$$\eta_g(x) = x + \sigma_g^2 \eta_g(0, 1). \tag{5}$$

As gradients need to flow through x to the meta-optic parameters, moving it outside of the stochastic function η_g permits differentiability. Differentiable Poisson noise is realized using the Score-Gradient trick[12], which we implement in TensorFlow 2 as follows:

```
import tensorflow_probability as tfp
p = tfp.distributions.Poisson(rate=x/a_p, validate_args=True)
sampled = tfp.monte_carlo.expectation(f=lambda z:z,
    samples=p.sample(1), log_prob=p.log_prob,
    use_reparameterization=False)
eta_p = sampled*a_p
```

Expressing sensor noise as a differentiable step within the image formation pipeline confers another advantage in that we do not require hand-engineered regularizers in our loss function, instead our neural nano-optic naturally learns how to accommodate for measurement error. Traditional hand-crafted regularizers such as total variation often blur out high frequency details. In addition to the descriptions provided in this document, we refer the reader to the public code repository.

Feature Space Deconvolution As described in the main manuscript, we employ a fully differentiable neural deconvolution method f_{DECONV} that utilizes a novel feature propagation scheme:

$$f_{\text{DECONV}}(\mathbf{I}) = f_{\text{DE}}(f_{\text{Z}\to\text{W}}(f_{\text{FE}}(\mathbf{I}))). \tag{6}$$

Each function component consists of optimizable parameters. Specifically, f_{FE} and f_{DE} both consist of convolutional neural networks (CNN) and their optimizable parameters $\mathcal{P}_{\text{FE}}$ and $\mathcal{P}_{\text{DE}}$ consist of neural network weights. The optimizable parameters $\mathcal{P}_{\text{Z}\to\text{W}}$ for $f_{\text{Z}\to\text{W}}$ depend on the choice of feature propagator, for example a Wiener filter $\mathcal{P}_{\text{Z}\to\text{W}}$ would consist of the SNR parameter. All of these parameters, given by $\mathcal{P}_{\text{DECONV}} = \mathcal{P}_{\text{FE}} \cup \mathcal{P}_{\text{Z}\to\text{W}} \cup \mathcal{P}_{\text{DE}}$, define our deconvolution algorithm. For an input image $\mathbf{I}$, the deconvolved output image $\mathbf{O}$ is given by

$$\mathbf{O} = f_{\text{DECONV}}(\mathbf{I}, \text{PSF}, \mathcal{P}_{\text{DECONV}}). \tag{7}$$

All of $\mathcal{P}_{\text{DECONV}}$ is jointly optimized together with $\mathcal{P}_{\text{META}}$ during our end-to-end design.

Spatially Varying Image Formation We simulate the spatially varying aberrations incurred by the PSFs in a patch-based manner. We first divide the FOV into an $M \times M$ grid of patches. Then we employ an offset PSF design procedure as described below:

1. Let PSF_θ be the PSF for a single patch of our $M \times M$ grid at field angle θ. Let $\text{PSF}_{\theta+\epsilon}$ be the PSF at field angle $\theta + \epsilon$, where ϵ is a small angle. $\text{PSF}_{\theta+\epsilon}$ is effectively the PSF at the outer periphery of a patch.

2. For the forward pass we use $\text{PSF}_{\theta+\epsilon}$ but for deconvolution we use PSF_θ. This process acts as a regularization to the PSF design, the variance between PSF_θ and $\text{PSF}_{\theta+\epsilon}$ cannot be too severe.

3. Repeat for other values of θ to cover the whole $M \times M$ grid. Compute the loss for each deconvolved patch individually and then backpropagate the total loss to all trainable parameters.

See Supplementary Fig. 4 for an illustration of this offset PSF procedure. For our experiments we set $M = 5$ and $\epsilon = 5°$.

After design and fabrication, we account for mismatches between the PSF simulated by our proxy model and the experimentally measured PSF by performing a PSF calibration step. See Supplementary Note 7 for PSF measurement details. We finetune our deconvolution algorithm using the measured PSFs.

Fully Differentiable Metasurface Imaging Altogether, our metasurface imaging pipeline allows us to apply first-order stochastic gradient optimization to jointly optimize for $\mathcal{P}_{\text{META}}$ and $\mathcal{P}_{\text{DECONV}}$ that will minimize our user-defined endpoint loss function $\mathcal{L}$. In our case, given an input RGB image $\mathbf{I}$ we want parameter values that will recover $\mathbf{I}$ with high fidelity. As such, our endpoint loss $\mathcal{L}$ will measure perceptual image quality between $\mathbf{I}$ and the recovered image $\mathbf{O}$, see Supplementary Note 6 for how we define this loss function. During the end-to-end design stage our image formation is defined using the previously described offset PSF field angle scheme

$$\text{PSF}_\theta = f_{\text{META}}(\theta, \mathcal{P}_{\text{META}}, \mathcal{C}_{\text{META}}),\ \text{PSF}_{\theta+\epsilon} = f_{\text{META}}(\theta+\epsilon, \mathcal{P}_{\text{META}}, \mathcal{C}_{\text{META}}) \tag{8}$$

$$\mathbf{O}_\theta = f_{\text{DECONV}}(f_{\text{SENSOR}}(\mathbf{I}_\theta * \text{PSF}_{\theta+\epsilon}, \mathcal{C}_{\text{SENSOR}}), \text{PSF}_\theta, \mathcal{P}_{\text{DECONV}})), \tag{9}$$

where $\mathbf{I}_\theta$ is the image patch of $\mathbf{I}$ corresponding to field angle θ and $*$ is the convolution operator. We then apply auto-differentiation optimizers to solve for the following

$$\{\mathcal{P}^*_{\text{META}}, \mathcal{P}^*_{\text{DECONV}}\} = \underset{\mathcal{P}_{\text{META}}, \mathcal{P}_{\text{DECONV}}}{\operatorname{argmin}} \sum_{i=1}^{M} \sum_{\theta} \mathcal{L}(\mathbf{O}^{(i)}_\theta, \mathbf{I}^{(i)}_\theta), \tag{10}$$

where M is the number of training samples. Once training is complete we use $\mathcal{P}^*_{\text{META}}$ to manufacture our meta-optic. We finetune $\mathcal{P}^*_{\text{DECONV}}$ using the measured PSFs of the fabricated meta-optic.

Summary of Parameters We summarize all parameters used in our image formation and deconvolution in Supplementary Table 5. Altogether, our pipeline optimizes two sets of parameters $\mathcal{P}_{\text{META}}$ and $\mathcal{P}_{\text{DECONV}}$. $\mathcal{P}_{\text{META}}$ consists of the parameters that determine the phase profile of the metasurface optic and $\mathcal{P}_{\text{DECONV}}$ are the parameters corresponding to the reconstruction algorithm. We also make use of experimentally measured or calibrated parameters that are fixed during the optimization. These include the polynomial coefficients $\mathcal{C}_{\text{META}}$ for metasurface proxy model and the sensor noise parameters $\mathcal{C}_{\text{SENSOR}}$.

Supplementary Table 3: Fitted polynomial coefficients for the inverse, phase-to-structure mapping.

Coefficient	b_0	b_1	b_2
Value	-0.1484	0.6809	0.2923

Supplementary Table 4: Fitted polynomial coefficients for the forward, structure-to-phase mapping. Note that c_{12}, c_{21}, c_{22} are all zero.

Coefficient	c_{00}	c_{01} (nm^{-1})	c_{10}	c_{02} (nm^{-2})	c_{11} (nm^{-1})	c_{20}
Value	6.051	-2.03×10^{-2}	2.26	1.37×10^{-5}	-2.95×10^{-3}	0.797

Supplementary Table 5: Summary of parameters used for image formation and deconvolution. We differentiate between optimizable and fixed parameters. See Supplementary Fig. 4 for an illustration of how these parameters are connected together.

Parameter Set	Parameters	Optimizable	Notes
$\mathcal{C}_{\text{META}}$	$\{b_0, b_1, b_2\}$ $\{c_{00}, c_{01}, c_{10}, c_{02}, c_{11}, c_{20}\}$	No	See Supplementary Tables 3 and 4.
$\mathcal{P}_{\text{META}}$	$\{a_0, \ldots, a_n\}$	Yes	See Eq. 1 in main manuscript.
$\mathcal{C}_{\text{SENSOR}}$	$\{a_p, \sigma_g\}$	No	Experimentally measured.
$\mathcal{P}_{\text{DECONV}}$	$\mathcal{P}_{\text{FE}} \cup \mathcal{P}_{\text{Z}\to\text{W}} \cup \mathcal{P}_{\text{DE}}$	Yes	$\mathcal{P}_{\text{FE}}$ and $\mathcal{P}_{\text{DE}}$ consist of neural network parameters while $\mathcal{P}_{\text{Z}\to\text{W}}$ consists of parameters specific to the feature propagator (e.g. the SNR parameter for a Wiener filter).

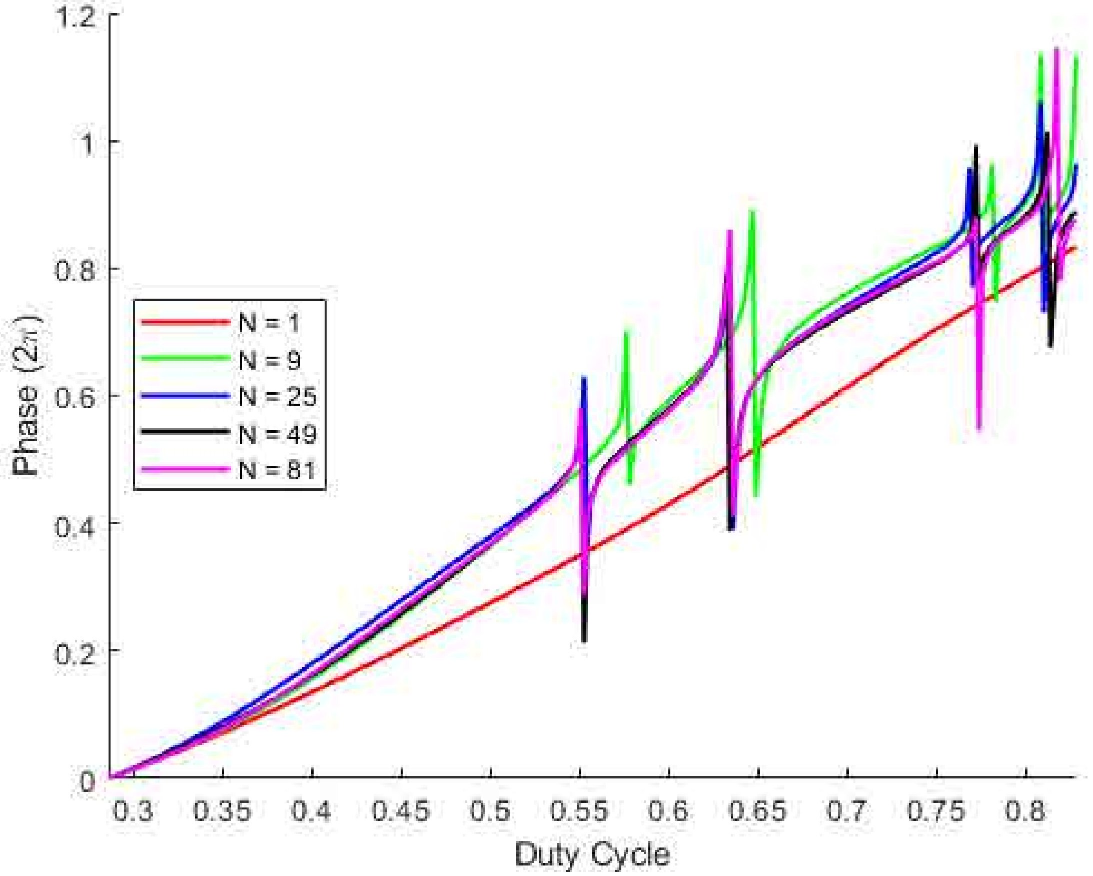

Supplementary Figure 2: Metasurface scatterer simulation. The transmission coefficent as a function of duty cycle was calculated using rigorous coupled-wave analysis. By considering the scatterer's permittivity with only the DC Fourier component, we neglect contributions from higher order Fourier terms but still capture an approximate transmission coefficient that closely follows the response with additional Fourier terms. Here, $N = 1$ denotes the effective index approximation-based transmission coefficient phase used in our design.

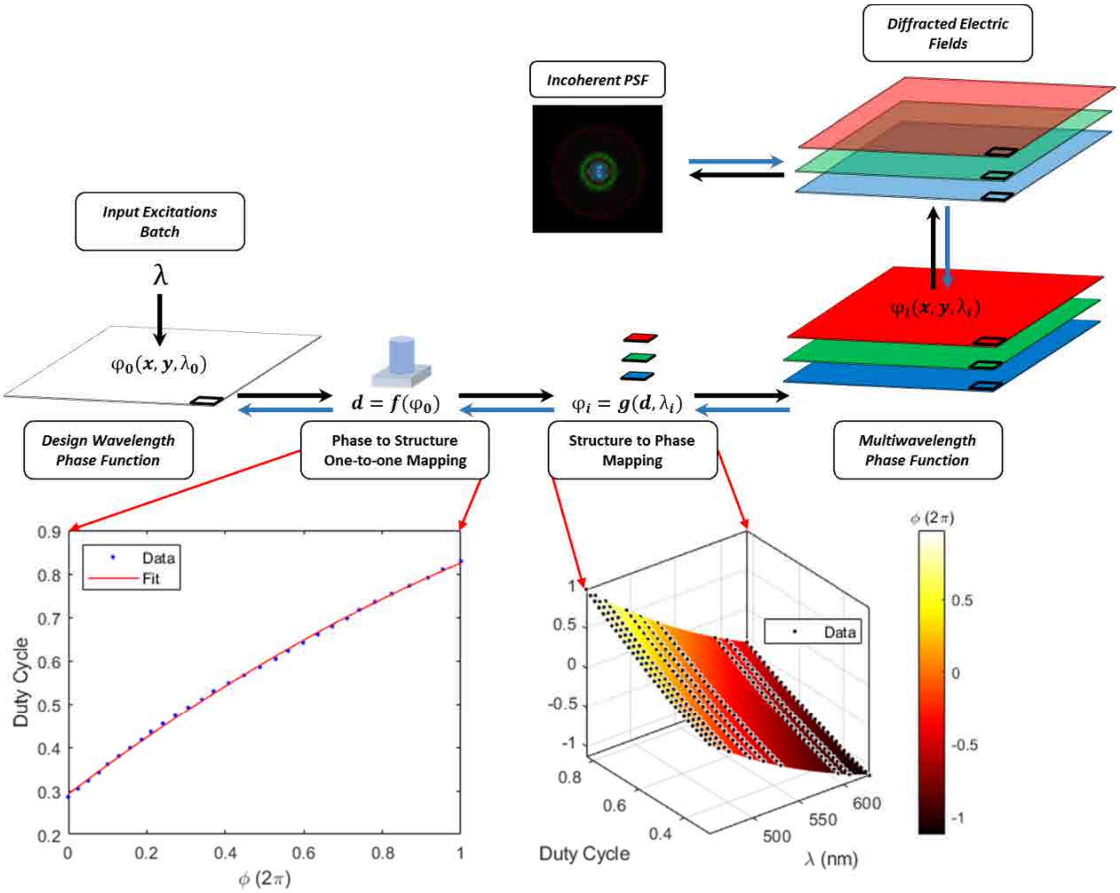

Supplementary Figure 3: Differentiable proxy-based metasurface simulation. To determine the metasurface phase at all simulated wavelengths, we sequentially apply differentiable inverse and forward phase mappings. From a desired phase at the nominal wavelength, we first compute the required scatterer distribution, mapping from phase to the structure in an inverse manner. With the structure defined, we then apply the forward mapping, converting the duty cycles into phases for the multiple wavelengths used in the end-to-end-design. With the phase at each wavelength determined, we then diffract the electric fields to the image plane and we can compute the incoherent PSF by taking the modulus squared.

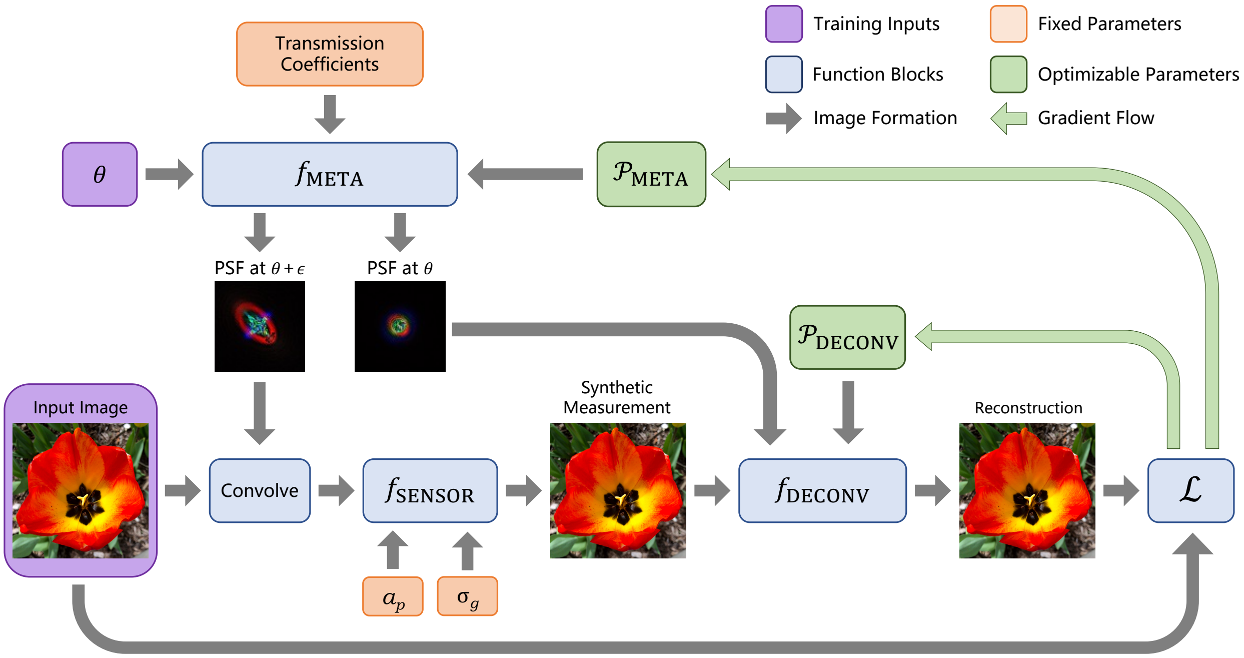

Supplementary Figure 4: Design stage image formation and deconvolution. We minimize PSF spatial variance within each patch during the end-to-end design stage by using $\text{PSF}_{\theta+\epsilon}$ for the image formation (forward pass) and PSF_{θ} for the non-blind deconvolution.

Supplementary Note 5: Neural Feature Propagation

Correcting for the severe chromatic aberrations incurred by a meta-optic requires a robust deconvolution algorithm. To tackle this problem, we design a deconvolution approach that exploits the advantages of both traditional deconvolution methods and deep neural networks. Traditional deconvolution methods, such as Wiener filtering or optimization-based deconvolution[13,14], rely on an analytical model of the image formation process. As a result, these methods are capable of deconvolving diverse aberrations and generalize well to unseen datasets – simply by adjusting the forward model parameters of the PSF and noise model. However, they often rely on naive regularizers such as $\mathcal{L}_1$ total variation[14,15]. In contrast, recent deep learning methods have demonstrated high-quality reconstruction results for specific datasets[16] but fail to generalize to experimental test datasets, including data with large PSFs of severely aberrated optics as we will show below.

We overcome the limitations of these two directions by combining learned feature extraction with model-based deconvolution that achieves three key outcomes: improved generalization compared to supervised deep learning methods, high-frequency reconstruction results, and robustness to sensor noise. As described in the main manuscript, we employ a neural feature propagation architecture that comprises three stages: a multi-scale feature extractor f_{FE}, feature propagation through inverse filters $f_{\text{Z}\rightarrow\text{W}}$ (where Z are extracted features and W are the corresponding deconvolved features), and multi-scale decoding f_{DE}. Formally, our feature propagation network performs the following:

$$\mathbf{O} = \underset{\substack{\uparrow \\ \text{Decoder}}}{f_{\text{DE}}} \left(\overset{\substack{\text{Feature Propagation} \\ \downarrow}}{f_{\text{Z}\rightarrow\text{W}}} \left(\underset{\substack{\uparrow \\ \text{Feature Extraction}}}{f_{\text{FE}}} (\mathbf{I}), \text{PSF} \right) \right), \tag{11}$$

where $\mathbf{I}$ is the raw sensor measurement and $\mathbf{O}$ is the output image. Any differentiable deconvolution method, where the outputs are differentiable with respect to the inputs, can be used for $f_{\text{Z}\rightarrow\text{W}}$. This includes blind deconvolution methods that do not utilize the PSF. We use Wiener filtering with edgetapering[17] to reduce boundary artifacts. An overview of our network architecture is shown in Supplementary Fig. 5 and an explicit description can be found in Supplementary Tables 6, 7, 8. Note that all stages f_{FE}, $f_{\text{Z}\rightarrow\text{W}}$, f_{DE} are jointly optimized together with the meta-optic parameters, so the SNR parameter that is used for Wiener filtering in $f_{\text{Z}\rightarrow\text{W}}$ is also optimized.

Our feature extractor f_{FE} employs a series of convolutional neural network (CNN) layers to determine image features at the original resolution and also at $2\times$ and $4\times$ downsampled resolutions. Performing feature extraction at lower resolutions allows the network to determine features at a global level while the images extracted at the original resolution focus on local details. To accommodate the different image resolutions, we bilinearly resize the PSF to $1\times$, $2\times$, $4\times$ downsampled resolutions before feeding to the feature propagator. The feature extractor also converts a 3-channel RGB image into feature tensors with 15, 30, 60 channels corresponding to $1\times$, $2\times$, $4\times$ resolution. We repeat our 3-channel RGB PSF to match the same number of channels as shown in Supplementary Fig. 5. After passing through $f_{\text{Z}\rightarrow\text{W}}$ the processed image tensors are combined by

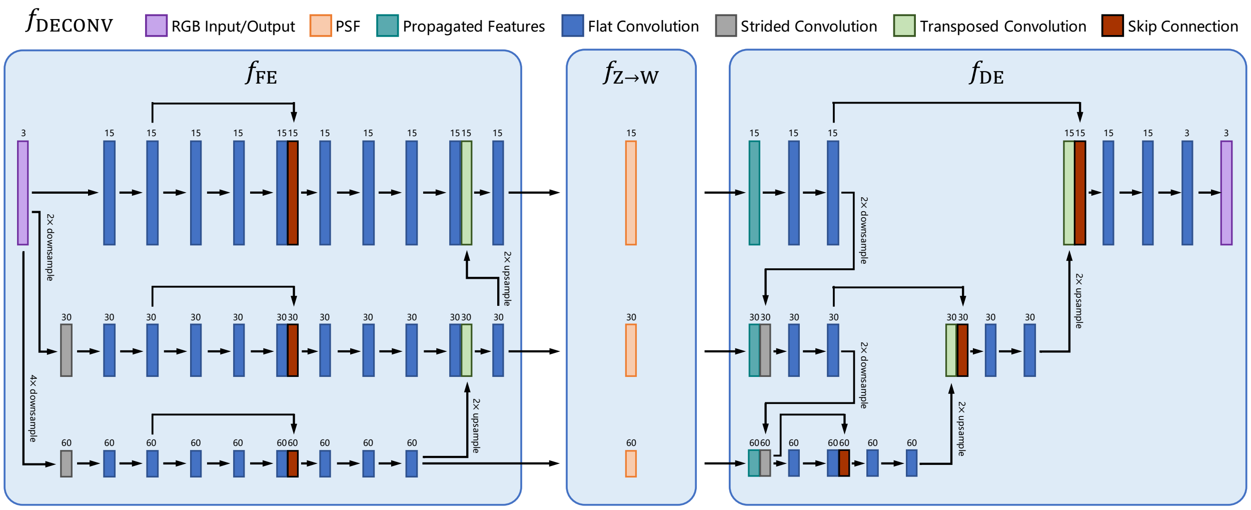

Supplementary Figure 5: Architecture of neural feature propagator network. The feature extractor f_{FE} uses CNNs to extract features at $1\times$, $2\times$, $4\times$ downsampled resolutions, which allows for learning of global and local features. The learned features are shared across the different resolutions. After extraction, the feature tensors are propagated through $f_{\text{Z}\rightarrow\text{W}}$. The PSF is used by the feature propagator for non-blind deconvolution, and the PSF is resized for different image resolutions. After feature propagation, the decoder f_{DE} again applies CNNs to fuse the feature tensors into a single RGB reconstruction. Our decoder also shares information across image resolutions. Skip connections (concatenation of previous feature tensors) are employed by both f_{FE} and f_{DE} to better shuttle image features across the network. In the figure, the number of feature channels is shown above the operation layers.

our decoder f_{DE}. f_{DE} also operates at multiple resolutions and progressively fuses global information obtained at smaller resolutions into higher resolution feature maps. All of the feature tensors are eventually processed into a single 3-channel RGB output image. We employ skip connections throughout f_{FE} and f_{DE} as they have been shown to improve image feature learning by shuttling information from earlier feature layers[18].

Supplementary Table 6: Neural feature propagator network architecture for the feature extractor. In the table, "conv-c(a)-k(b)-s(c)-LRelu" represents a convolution layer with a output channels, a $b \times b$ kernel window, using stride c, followed by a Leaky Relu ($\alpha = 0.02$) activation function. We use convT to denote transposed convolution.

**Neural Feature Propagator (Feature Extractor f_{FE})**		
Layer Name	Layer Type	Input Layer
Input	RGB	
down_l0	conv-c15-k7-s1-LRelu	Input
down_l0	conv-c15-k7-s1-LRelu	down_l0
down_l1	conv-c30-k5-s2-LRelu	down_l0
down_l1	conv-c30-k3-s1-LRelu	down_l1
down_l1	conv-c30-k3-s1-LRelu	down_l1
down_l2	conv-c60-k5-s2-LRelu	down_l1
down_l2	conv-c60-k3-s1-LRelu	down_l2
down_l2	conv-c60-k3-s1-LRelu	down_l2
conv_l2_k0	conv-c60-k3-s1-LRelu	down_l2
conv_l2_k1	conv-c60-k3-s1-LRelu	conv_l2_k0
conv_l2_k2	conv-c60-k3-s1-LRelu	Concat([conv_l2_k0, conv_l2_k1])
conv_l2_k3	conv-c60-k3-s1-LRelu	conv_l2_k2
conv_l2_k4	conv-c60-k3-s1-LRelu	conv_l2_k3
conv_l2_k5	conv-c60-k3-s1-LRelu	conv_l2_k4
conv_l1_k0	conv-c30-k3-s1-LRelu	down_l1
conv_l1_k1	conv-c30-k3-s1-LRelu	conv_l1_k0
conv_l1_k2	conv-c30-k3-s1-LRelu	Concat([conv_l1_k0, conv_l1_k1])
conv_l1_k3	conv-c30-k3-s1-LRelu	conv_l1_k2
conv_l1_k4	conv-c30-k3-s1-LRelu	conv_l1_k3
conv_l1_k5	conv-c30-k3-s1-LRelu	conv_l1_k4
up_l2	convT-c30-k2-s2-LRelu	conv_l2_k5
conv_l1_k6	conv-c30-k3-s1-LRelu	Concat([up_l2, conv_l1_k5])
conv_l1_k7	conv-c30-k3-s1-LRelu	conv_l1_k6
conv_l0_k0	conv-c15-k3-s1-LRelu	down_l0
conv_l0_k1	conv-c15-k3-s1-LRelu	conv_l0_k0
conv_l0_k2	conv-c15-k3-s1-LRelu	Concat([conv_l1_k0, conv_l0_k1])
conv_l0_k3	conv-c15-k3-s1-LRelu	conv_l0_k2
conv_l0_k4	conv-c15-k3-s1-LRelu	conv_l0_k3
conv_l0_k5	conv-c15-k3-s1-LRelu	conv_l0_k4
up_l1	convT-c15-k2-s2-LRelu	conv_l1_k5
conv_l0_k6	conv-c15-k3-s1-LRelu	Concat([up_l1, conv_l0_k5])
conv_l0_k7	conv-c15-k3-s1-LRelu	conv_l0_k6

Supplementary Table 7: Neural feature propagator network architecture for the feature propagator. This network takes in layers from the feature extractor as input.

**Neural Feature Propagator (Feature Propagator $f_{\text{Z}\to\text{W}}$)**		
Layer Name	Layer Type	Input Layer
PSF_1x	RGB PSF	
PSF_2x	$2\times$ downsampled RGB PSF	
PSF_4x	$4\times$ downsampled RGB PSF	
fp_l0	Feature Propagator	PSF_1x and conv_l0_k7
fp_l1	Feature Propagator	PSF_2x and conv_l1_k7
fp_l2	Feature Propagator	PSF_4x and conv_l2_k5

Supplementary Table 8: Neural feature propagator network architecture for the decoder. In the table, "conv-c(a)-k(b)-s(c)-LRelu" represents a convolution layer with a output channels, a $b \times b$ kernel window, using stride c, followed by a Leaky Relu ($\alpha = 0.02$) activation function. We use convT to denote transposed convolution. This network takes in layers from the feature propagator as input.

**Neural Feature Propagator (Decoder f_{DE})**		
Layer Name	Layer Type	Input Layer
conv_l0_k0	conv-c30-k5-s1-LRelu	fp_l0
conv_l0_k1	conv-c30-k5-s1-LRelu	conv_l0_k0
down_l0	conv-c30-k5-s2-LRelu	conv_l0_k1
conv_l1_k0	conv-c60-k3-s1-LRelu	Concat([fp_l1, down_l0])
conv_l1_k1	conv-c60-k3-s1-LRelu	conv_l1_k0
down_l1	conv-c60-k3-s2-LRelu	conv_l1_k1
conv_l2_k0	conv-c120-k3-s1-LRelu	Concat([fp_l2, down_l1])
conv_l2_k1	conv-c120-k3-s1-LRelu	conv_l2_k0
conv_l2_k2	conv-c120-k3-s1-LRelu	Concat([conv_l2_k0, fp_l2, down_l1])
conv_l2_k3	conv-c120-k3-s1-LRelu	conv_l2_k2
up_l2	convT-c60-k2-s2-LRelu	conv_l2_k3
conv_l1_k2	conv-c60-k3-s1-LRelu	Concat([conv_l1_k1, up_l2])
conv_l1_k3	conv-c60-k3-s1-LRelu	conv_l1_k2
up_l1	convT-c60-k2-s2-LRelu	conv_l2_k3
conv_l0_k2	conv-c30-k5-s1-LRelu	Concat([conv_l0_k1, up_l1])
Output	RGB	conv_l0_k2

Supplementary Note 6: Joint Optimization Method

Our endpoint loss function is designed for perceptual image quality. To that end, we leverage a combination of objectives. The loss function is given by

$$\mathcal{L} = \lambda_1 \mathcal{L}_1 + \lambda_{\text{perc}} \mathcal{L}_{\text{perc}} + \lambda_{\text{grad}} \mathcal{L}_{\text{grad}}, \tag{12}$$

where $\mathcal{L}_1$ is the Mean Absolute Error function, $\mathcal{L}_{\text{perc}}$ is a VGG-19 based perceptual loss function[19], $\mathcal{L}_{\text{grad}}$ is a spatial gradient loss, and $\{\lambda_1, \lambda_{\text{perc}}, \lambda_{\text{grad}}\}$ are loss weighting coefficients.

Recent advances in deep learning have demonstrated that computing differences on extracted feature maps[20] acts as a robust measure for image similarity with respect to human visual judgment, in contrast to traditional metrics such as mean squared error (MSE) and Structural Similarity[21]. As such, we leverage these insights in our loss function through our perceptual loss component $\mathcal{L}_{\text{perc}}$ which uses a pre-trained VGG-19 network to extract and compare features from the output RGB image I_{out} and the ground truth RGB image I_{gt}:

$$\mathcal{L}_{\text{perc}}(I_{\text{out}}, I_{\text{gt}}) = \sum_{b=2,3} \mathcal{L}_1 \left(\Phi_{b,2}(I_{\text{out}}), \Phi_{b,2}(I_{\text{gt}}) \right), \tag{13}$$

where $\Phi_{b,2}$ is the extracted feature map at layer block$b$_conv2 of the VGG-19 network.

While the perceptual loss significantly improves overall image quality reconstructions, we did notice that fine details were still missing from the final output. We attribute this to computing losses on extracted feature maps with coarser resolution, as these lower resolution feature maps no longer are sensitive to fine differences at the original higher resolution. To remedy this, we also leverage a spatial gradient loss $\mathcal{L}_{\text{grad}}$. To compute the gradient loss we first define the following four kernels:

$$k_1 = \begin{bmatrix} 1 & -1 \end{bmatrix}, k_2 = \begin{bmatrix} 1 \\ -1 \end{bmatrix}, k_3 = \begin{bmatrix} -1 & 0 \\ 0 & 1 \end{bmatrix}, k_4 = \begin{bmatrix} 0 & 1 \\ -1 & 0 \end{bmatrix}. \tag{14}$$

We then compute $\mathcal{L}_{\text{grad}}$ as

$$\mathcal{L}_{\text{grad}} = \sum_{n=1,2,3,4} \mathcal{L}_1(I_{\text{out}} * k_n, I_{\text{gt}} * k_n), \tag{15}$$

where $*$ is the convolution operator. In addition to the descriptions provided in this document, we refer the reader to the public code repository.

We optimize the entire end-to-end pipeline in TensorFlow 2, and the entire optimization procedure took 18 hours on a single Nvidia P100 GPU. During training, we employ an alternating optimization scheme where we alternate between optimizing the meta-optic's phase coefficients and optimizing the neural deconvolution. Specifically, we employ the Adam optimizer[22] and we alternate between optimizing the meta-optic phase for $n_{\text{META}} = 10$ iterations with learning rate $50\times$

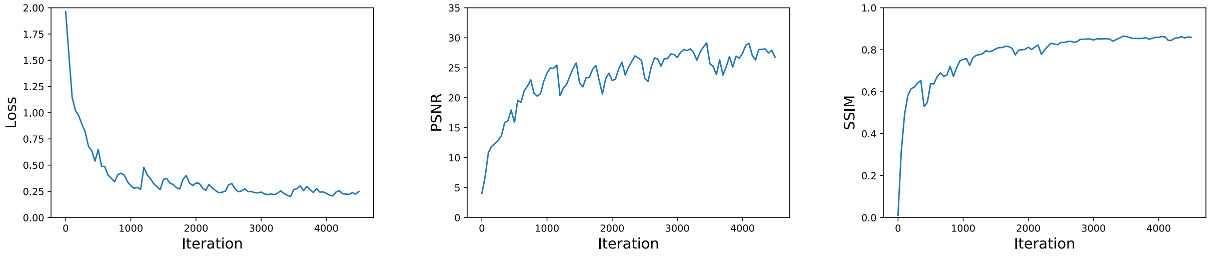

Supplementary Figure 6: Progression of training loss function and perceptual metrics during optimization.

10^{-4} and optimizing the neural deconvolution for $n_{\text{DECONV}} = 100$ iterations with learning rate 1×10^{-4}. Secondly, as the meta-optic phase coefficients are unbounded we restrict the parameter range for all phase coefficients to be within $[-1000, 1000]$ and during optimization we normalize the range to be within $[-1, 1]$. The metasurface phase parameters are initialized at zero and the neural network parameters for our deconvolution network are randomly initialized and are unbounded. The SNR parameter for our inverse filtering block is expressed as 10^{α} and α is bounded within $[3, 5]$, we initialize $\alpha = 4$. See Supplementary Algorithm 1 and the public code repository for the full algorithm. The final optimized phase coefficients that we obtained are given in Supplementary Table 9. For training we use images from the INRIA Holiday dataset[23].

We emphasize that our deconvolution algorithm generalizes to unseen experimental captures despite never having seen these images. All training and finetuning is performed solely in simulation, and we only use the measured PSFs to account for any discrepancies between theoretical prediction and empirical observation. The generalizability of the neural feature propagation algorithm to experimental measurements is shown in the main manuscript and in Supplementary Note 11. Specifically, the high-quality reconstructions of natural scenes such as the fruits in Fig. 2 of the main manuscript, the sea shore in the third row of Supplementary Fig. 20, the urban area in the middle row of Supplementary Fig. 21, the newspaper in the middle row of Supplementary Fig. 22, and the toys in the middle row of Supplementary Fig. 23, as well as of the USAF 1951 and Siemens Star charts in Supplementary Figs. 17 and 18 are all achieved with our neural nano-optics *even though the reconstruction algorithm never saw the sensor measurement or the corresponding ground truth during training*.

We show metric quantities over the design optimization process in Supplementary Fig. 6. We observe improving performance over the course of optimization, which validates that our optimization procedure is indeed converging upon a desirable solution.

Supplementary Table 9: Final optimized metasurface phase coefficients. These phase coefficients were obtained with our end-to-end pipeline and were used to manufacture the meta-optic.

Phase Coefficient	a_2	a_4	a_6	a_8	a_{10}	a_{12}	a_{14}	a_{16}
Value	-349.48642	-3.2419178	-1000	-1000	-1000	-1000	-1000	-1000

Supplementary Algorithm 1: Neural Nano-Optics Design Algorithm

$\{\mathcal{C}_{\text{META}}, \mathcal{C}_{\text{SENSOR}}\} =$ `Define_Fixed_Parameters()`

$\{\mathcal{P}_{\text{META}}, \mathcal{P}_{\text{DECONV}}\} =$ `Initialize_Optimizable_Parameters()`

$\mathcal{L} = \lambda_1 \mathcal{L}_1 + \lambda_{\text{perc}} \mathcal{L}_{\text{perc}} + \lambda_{\text{grad}} \mathcal{L}_{\text{grad}}$

for $t = 1, \ldots, n$ **do** `// Alternating Optimization`

  $\mathbf{I} =$ `RandomImage()`

  $\theta =$ `Field_Angle`

  $\text{PSF}_{\text{conv}} = f_{\text{META}}(\theta + \epsilon, \mathcal{P}_{\text{META}}, \mathcal{C}_{\text{META}})$

  $\text{PSF}_{\text{deconv}} = f_{\text{META}}(\theta, \mathcal{P}_{\text{META}}, \mathcal{C}_{\text{META}})$

  $\mathbf{O} = f_{\text{DECONV}}(f_{\text{SENSOR}}(\mathbf{I} * \text{PSF}_{\text{conv}}, \mathcal{C}_{\text{SENSOR}}, \mathcal{P}_{\text{META}}), \text{PSF}_{\text{deconv}}, \mathcal{P}_{\text{DECONV}})$

  for $j = 1, \ldots, n_{\text{META}}$ **do**

    `Update` $\left(\mathcal{P}_{\text{META}}, \partial \mathcal{L}(\mathbf{O}, \mathbf{I}) / \partial \mathcal{P}_{\text{META}}\right)$

    `Bound` $(\mathcal{P}_{\text{META}})$

  for $j = 1, \ldots, n_{\text{DECONV}}$ **do**

    `Update` $\left(\mathcal{P}_{\text{DECONV}}, \partial \mathcal{L}(\mathbf{O}, \mathbf{I}) / \partial \mathcal{P}_{\text{DECONV}}\right)$

    `Bound` $(\mathcal{P}_{\text{DECONV}})$

Supplementary Note 7: Experimental Setup

PSF Calibration Setup To measure the PSF, we imaged a pinhole positioned at the object plane that was backside illuminated by a fiber-coupled LED using the setup shown in Supplementary Fig. 7. As the sensor contains a cover-glass that is challenging to remove, we employ an optical relay system to mount the optic at a virtual distance of $500\,\mu m$ in front of our sensor. Specifically, the focal profile of the metasurface was relayed and magnified using a custom microscope comprising a $10\times$ Nikon objective and tube lens, projecting the image onto a Prosilica GT1930C Visible RGB camera. To measure the PSF at different field angles the pinhole and light source were mounted on an XY translation stage by using a pair of orthogonally oriented Thorlabs PT1 translation stages. The sensor pixel pitch of our camera is $5.86\,\mu m$ and our reconstructed images are $720\,px \times 720\,px$. The microscope was mounted on a Newport ILS series motorized linear stage to facilitate longitudinal positioning of the microscope. We acquire the PSFs over a range of exposure times ($0.002\,s$ and doubling up to and including $65.536\,s$) and then combine using HDR stitching. Additionally, each frame is computed from the average of 3 individual captures to reduce the noise present during PSF calibration and from this we subtract an average dark frame.

Imaging Setup The setup for image acquisition was identical to the PSF measurement setup except that a 5.5” SmallHD OLED display was used in place of the pinhole and the camera exposure time was adjusted to utilize the full dynamic range of the camera, see Supplementary Fig. 7. Again, we use the microscope relay to avoid having to remove the sensor cover glass. We use a display for scene capture to allow for a fair and reproducible scene capture process for comparing all meta-optics prototypes. For automated data collection, the Allied Vision Vimba API controlled the camera and we used an OpenCV-based Python script for loading images onto the OLED display. Unlike PSF acquisition, we only acquire a total of three images at $2\,s, 4\,s$, $6\,s$, $8\,s$ and we do not acquire multiples of the same frame. Note that the long exposure times are due to the large decrease in light intensity caused by our relay setup utilizing a $10\times$ magnification. Deploying the nano-optic without the magnifying objective and using a correspondingly sized sensor would require much shorter exposure times. Our $10\times$ magnification factor causes a $\sim 100\times$ decrease in light efficiency, and thus we expect to require $\sim 100\times$ shorter exposure times without the relay setup.

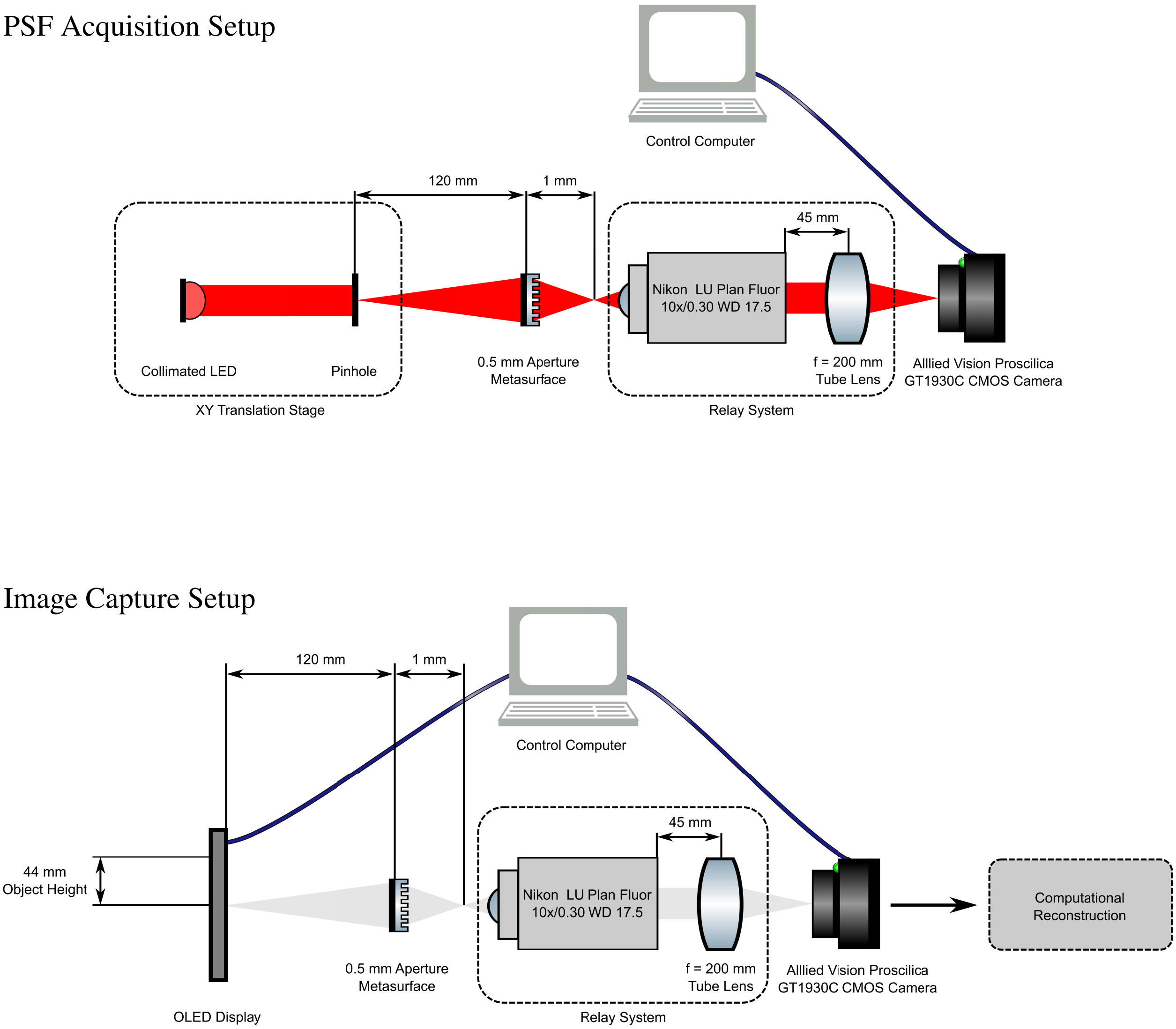

Supplementary Figure 7: Experimental imaging setup. We capture PSFs by using a movable pinhole and a laser source, with one laser for each of the RGB wavelengths. Once captured, the measured PSFs are used in our deconvolution algorithm and replace the simulated PSFs. We use the same setup for acquiring images except that the pinhole and lasers are replaced with a monitor that displays images.

Supplementary Note 8: Validation of Neural Nano-Optic Design

Existing Meta-Optics Designs We compare against existing metasurface designs and we demonstrate that our end-to-end optimized neural meta-optics design possesses significant advantages that facilitate deconvolution for image reconstruction. Specifically, we compare against a cubic[3], log-asphere[24], shifted axicon[25], and hyperboloid meta-optic engineered for green wavelengths ($511\,\mathrm{nm}$). These previously proposed meta-optics designs have been demonstrated before for full-color imaging applications; however, we demonstrate that using our proposed meta-optic design outperforms all of these existing approaches.

We now describe each of the existing metasurface designs. All meta-optics designs including our proposed design use a phase mask radius of $R = 250\,\mu\mathrm{m}$, corresponding to a $500\,\mu\mathrm{m}$ aperture. Furthermore, for all optics, except for the hyperboloid, we use a focal length of $f_0 = 1\,\mathrm{mm}$ and a nominal wavelength of $\lambda_0 = 452\,\mathrm{nm}$. This wavelength is selected as it is the center wavelength of the smallest wavelength (blue light) source in our OLED display and the phase at this wavelength exhibits a full 0 to 2π phase shift over the nanopost width range.

The phase relationship for the cubic meta-optic from Colburn et al.[3] is given by

$$\phi(x, y) = \frac{2\pi}{\lambda}\left(\sqrt{x^2 + y^2 + f^2} - f\right) + \frac{\alpha}{R^3}(x^3 + y^3), \tag{16}$$

where x and y are the plane coordinates and α is a design parameter that determines the strength of the cubic term. We set $\alpha = 86\pi$, $f = f_0$, and $\lambda = \lambda_0$. This α is set to be consistent with the prior work by Colburn et al.[3] but adjusted so that the design was scaled to the larger aperture size and focal length used in our current system. As the wavelength changes, a chromatic focal shift is induced for a metasurface and the α parameter is set to extend the depth of focus in a manner to compensate for the level of defocus across the desired operating bandwidth.

We compare against a log-asphere meta-optic from Chi et al.[24] with phase relationship given by

$$\phi(r) = \int_0^r \frac{r'}{\left(r'^2 + \left(s_1 + (s_2 - s_1)\left(\frac{r'}{R}\right)^n\right)^2\right)^{1/2}} dr', \tag{17}$$

where $r = \sqrt{x^2 + y^2}$, s_1 is the focal length of the central annular zone, s_2 is the focal length of the outermost annular zone, and n is a design parameter that affects the intensity distribution over the optical axis. In our experiments we set $n = 2$, making the intensity distribution uniform across the line of foci. We set $s_1 = 0.9f_0$ and $s_2 = 1.4f_0$ so that the longitudinal extension of the focal spot results in light focusing onto the sensor plane across the full operating bandwidth. Specifically, s_1 and s_2 are set asymmetrically as our nominal design wavelength ($452\,\mathrm{nm}$) is not at the center of our desired wavelength range.

The phase mask for the shifted axicon meta-optic from Wang et al.[25] follows the same form

as Eq. 17, except that we set $n = 1$ while keeping $s_1 = 0.9f_0$ and $s_2 = 1.4f_0$, which is the only difference between this design and the log-asphere.

Lastly, we compare against a hyperboloid meta-optic with phase relationship given by

$$\phi(x, y) = \frac{2\pi}{\lambda}\left(f - \sqrt{x^2 + y^2 + f^2}\right), \tag{18}$$

where we set $\lambda = 511\,\text{nm}$ to be focused at the green wavelength. We set $f = f_0 \cdot \lambda/\lambda_0$ to produce a meta-optic that is focused for a wavelength that is different from the nominal wavelength.

Meta-Optics Design Assessment We validate the proposed design and reconstruction method in simulation and experimentally. Characterizations of the meta-optics, including PSFs, MTFs, optical axis intensity measurements, and efficiency plots, are shown in Supplementary Figs. 11, 12, 13, 14. Note that all meta-optics designs for the comparison experiments in this note use a $500\,\mu\text{m}$ aperture. Colburn et al. [3] used a $200\,\mu\text{m}$ for the cubic meta-optic in their work, and in this Supplemental Document we report results for their a scaled $500\,\mu\text{m}$ variant. To scale their design to a $500\,\mu\text{m}$, f/2 aperture, we increased α such that it would equivalently compensate the number of waves of defocus imparted by sweeping the wavelength across the operating bandwidth from 400 to $700\,\text{nm}$.

Comparing all evaluated designs, we observe that the learned end-to-end optimized meta-optic design exhibits compact PSFs across the visible spectrum which allows for improved full-color deconvolution. Furthermore, the spatial variation of the PSFs across field angles is less severe than for the other meta-optic designs, which we owe to our spatial variation training scheme.

For synthetic evaluation, we compare the sensitivity of each optical design to spatial aberrations by using the same offset PSF field angle scheme that we employed for end-to-end design. Specifically, we use the PSF at field angle θ for deconvolution and the PSF at field angle $\theta + 5^\circ$ for image formation (forward pass). This scheme is applied to all meta-optics designs with the exception of the cubic for which we use normal incidence for both image formation and deconvolution. For fair comparison, we finetune our proposed neural deconvolution algorithm to each optical design (keeping the design fixed) and we compute quantitative metrics on an unseen test set, see Supplementary Table 10 for quantitative results and Supplementary Fig. 8 for qualitative results. We observed that our end-to-end optimized design enables the highest perceptual image quality and lowest reconstruction error.

For experimental evaluation, we manufacture each of the existing optical designs and perform imaging experiments. Qualitative reconstruction results for each meta-optic design using neural feature propagation deconvolution are shown in Supplementary Fig. 9 and the corresponding sensor measurements are shown in Supplementary Fig. 10. For the log-asphere, shifted axicon,

and hyperboloid meta-optics we observe that the spatial and wavelength sensitivity of these designs significantly reduces image quality. Although the cubic PSF exhibits spatial and wavelength invariance, the long PSF tails are challenging to deconvolve and leave post-deconvolution artifacts. The reconstructions for the shifted axicon and log-asphere designs exhibit strong ringing artifacts which can be especially noticed in the red channel, but also exist for the other color channels. The reconstructions for the traditional hyperboloid meta-optic exhibit sharp focus only for the target wavelength, with severe ringing for the other color channels. Our neural nano-optic is the only design that allows for high-fidelity reconstruction without cross channel artifacts.

Turning to the optical axis intensity measurements in Supplementary Fig. 13, we obtain further insights into the advantages of our learned nano-optic design. Our learned nano-optics maintains consistent aberration behavior across all wavelengths and field angles. In contrast, other designs such as the log-asphere, shifted-axicon, cubic, and hyperboloid undergo significant variation across wavelengths and the FOV. Furthermore, our design concentrates light for all wavelengths near the focal distance at 1 mm whereas designs such as the hyperboloid are only in focus for a single wavelength, even at normal incidence. These intensity measurements reveal that the optimization procedure steers the meta-optic design towards consistent, small aberration performance across wavelengths and the FOV.

The focusing efficiency plots in Supplementary Fig. 14 demonstrate additional insights. Here, we define the focusing efficiency as the ratio of the enclosed power at the focal plane relative to the incident power on the lens, where we sweep the enclosing radius across the size of the aperture. We observed that our learned design exhibits a slightly reduced focusing efficiency, showing that focusing efficiency is not a necessary constraint for the design of meta-optics for high-quality imaging. Nevertheless, this constraint can be easily incorporated into our optimization procedure and we show that this leads to higher focusing efficiency in Supplementary Fig. 14.

Supplementary Table 10: Quantitative comparison between different meta-optics designs in simulation. To evaluate the sensitivity to spatial aberrations we offset the field angle used for forward image formation and the field angle used for deconvolution. For a fair comparison, we apply our proposed neural feature propagation deconvolution for all designs, hence we achieve low reconstruction error even with imperfect meta-optic designs. Our end-to-end optimized meta-optics design allows for the lowest reconstruction error and highest PSNR. While some alternative designs preserve slightly sharper structure, which results in a higher SSIM, they forego color accuracy which acts as a detriment to the reconstruction error.

Meta-Optics Design	Image Formation PSF Field Angle θ_{conv}	Deconvolution PSF Field Angle θ_{deconv}	MSE	PSNR (dB)	SSIM
Neural Nano-Optics	$5°$	$0°$	**0.00185**	**27.7**	0.839
	$15°$	$10°$	**0.00273**	**26.1**	0.793
Cubic [3]	$5°$	$0°$	0.00352	24.9	0.728
	$15°$	$10°$	0.00388	24.5	0.710
Log-Asphere [24]	$5°$	$0°$	0.00217	27.3	0.811
	$15°$	$10°$	0.00395	24.7	0.748
Shifted Axicon [25]	$5°$	$0°$	0.00277	25.8	0.836
	$15°$	$10°$	0.00363	24.7	0.805
Hyperboloid (511 nm)	$5°$	$0°$	0.00249	27.4	**0.921**
	$15°$	$10°$	0.00315	26.0	**0.871**

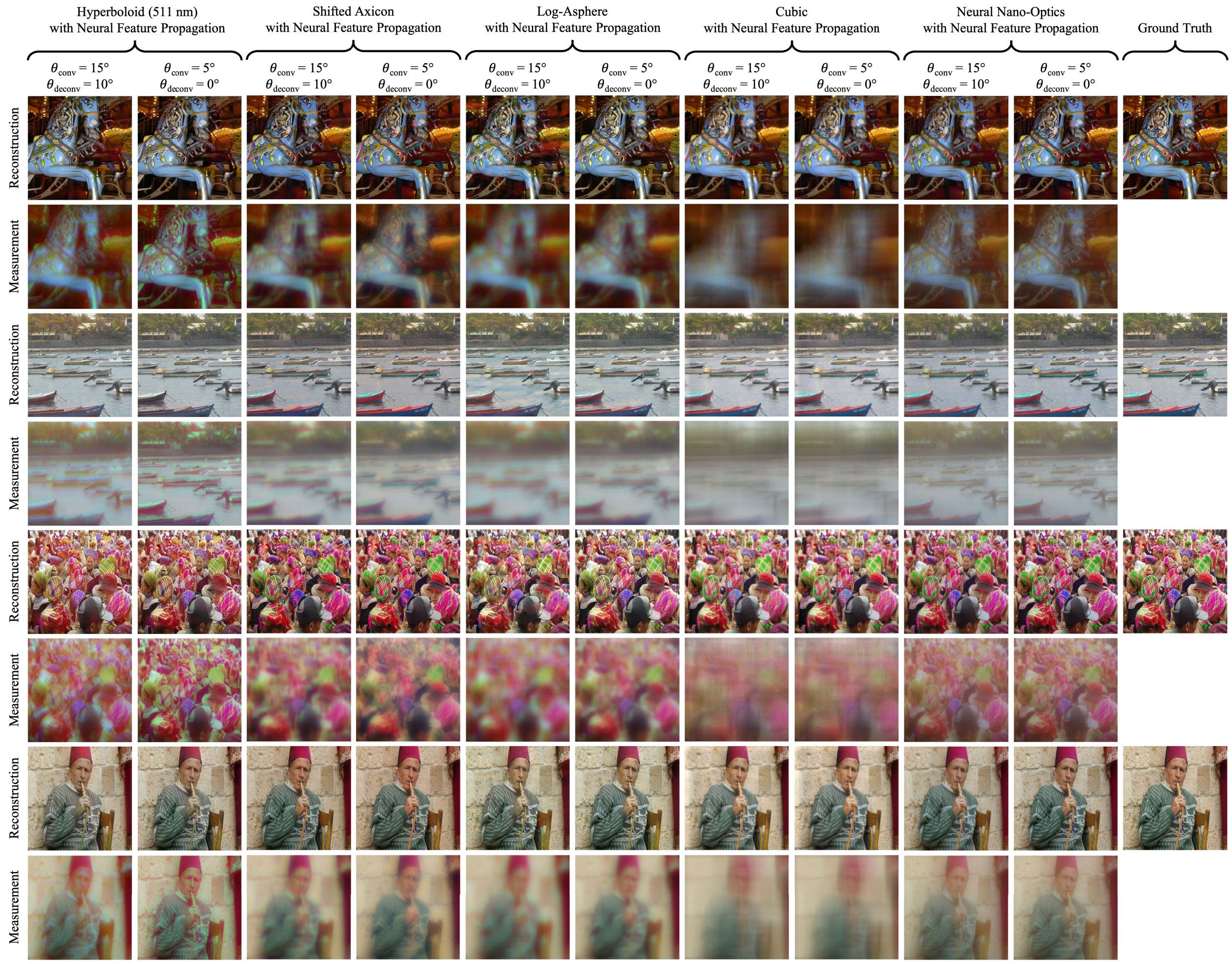

Supplementary Figure 8: Qualitative comparison for different meta-optics designs in simulation. For a fair comparison, we apply our proposed neural feature propagation deconvolution for all designs, hence we achieve high reconstruction qualities even with non-optimal meta-optic designs. Our optimized meta-optic produces the lowest image reconstruction error, as evidenced by fine image details and accurate color recovery. The aberrations from the cubic leave behind post-deconvolution artifacts. The log-asphere and shifted axicon meta-optics suffer from aberrations at higher field angles. While the hyperboloid has strong focus for the green channel which preserves spatial structure, it loses a significant amount of color information which negatively impacts downstream deconvolution.

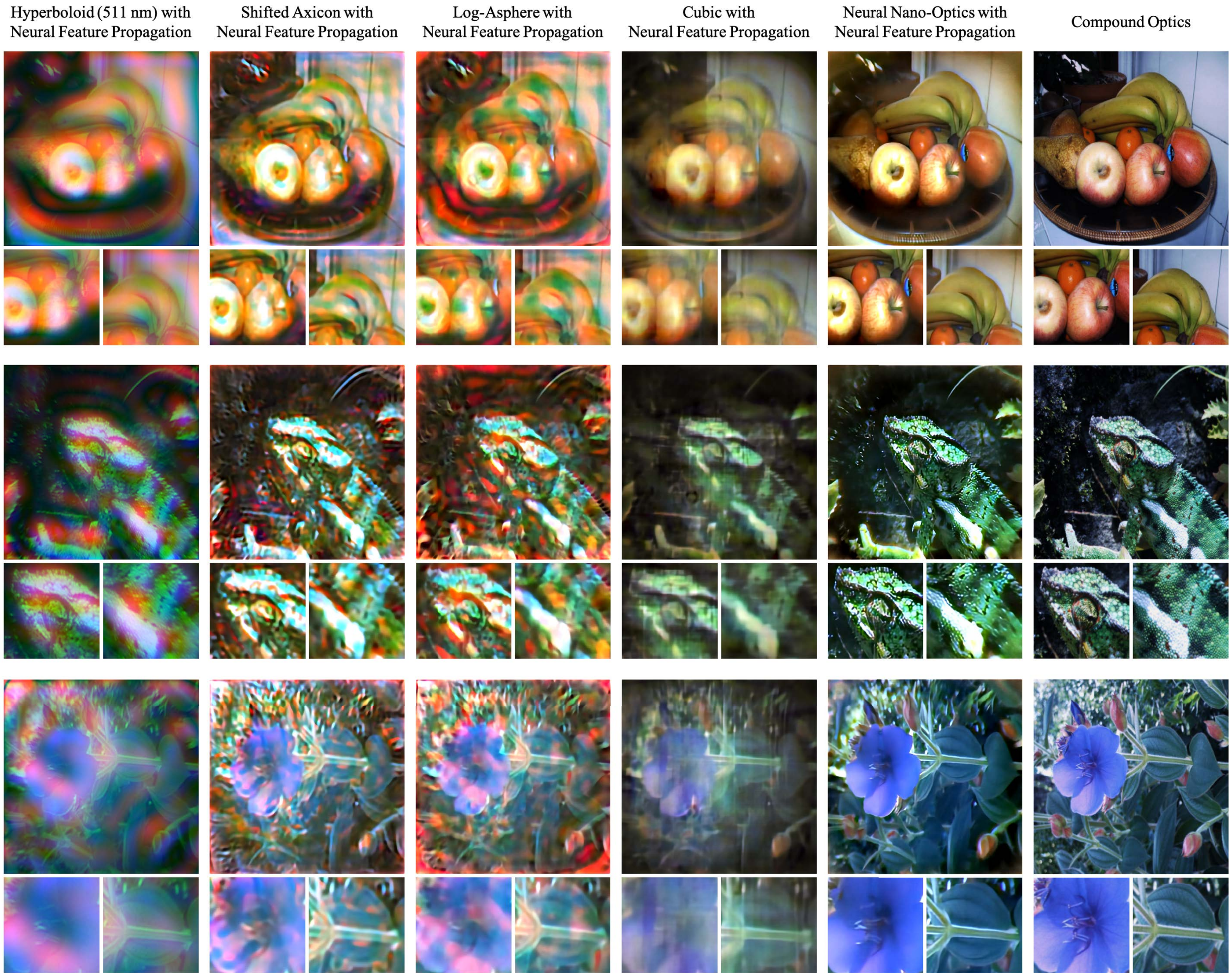

Supplementary Figure 9: Qualitative comparison for different meta-optics designs using experimental captures. The aberrations of the baseline meta-optics designs are too severe to allow for high-quality full-color imaging, although our proposed neural feature propagation does manage to recover some detail with these designs. The end-to-end optimized design is the only design that successfully facilitates downstream deconvolution.

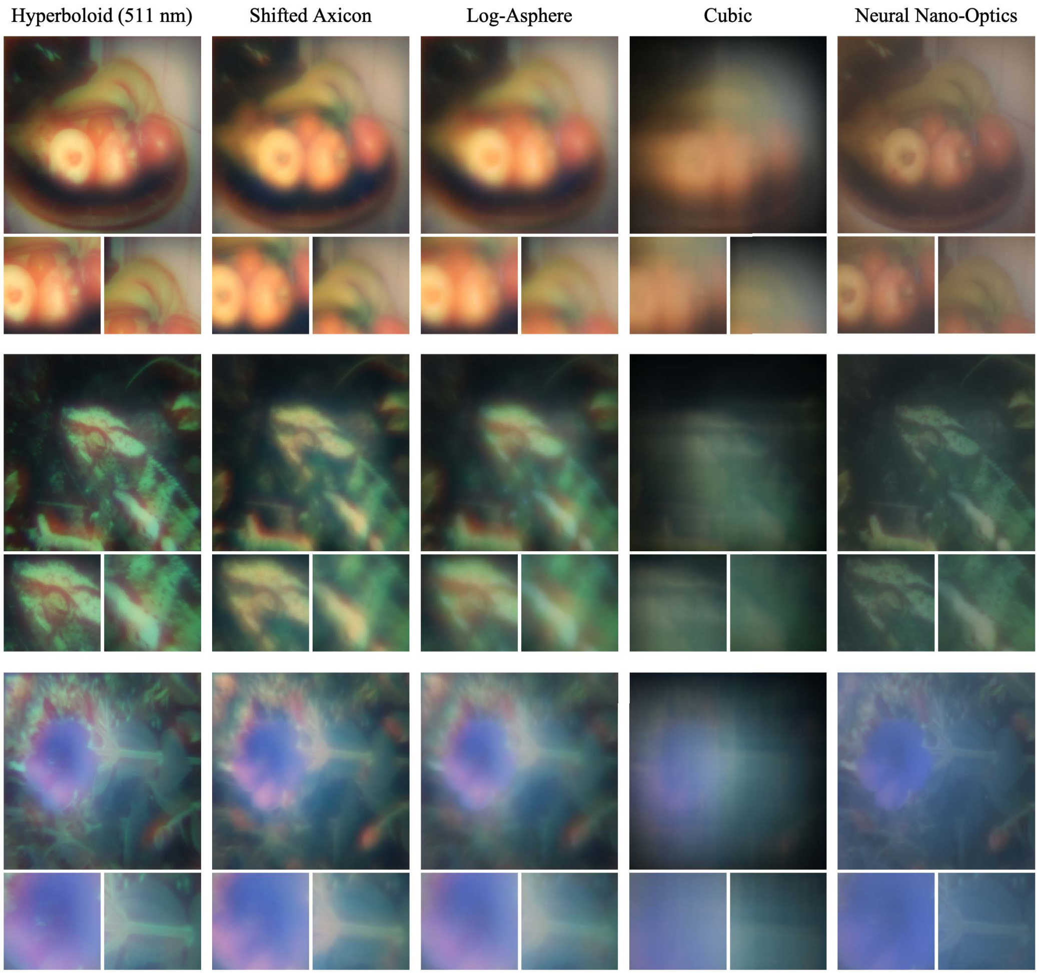

Supplementary Figure 10: Sensor measurements for the reconstructions in Supplementary Fig. 9.

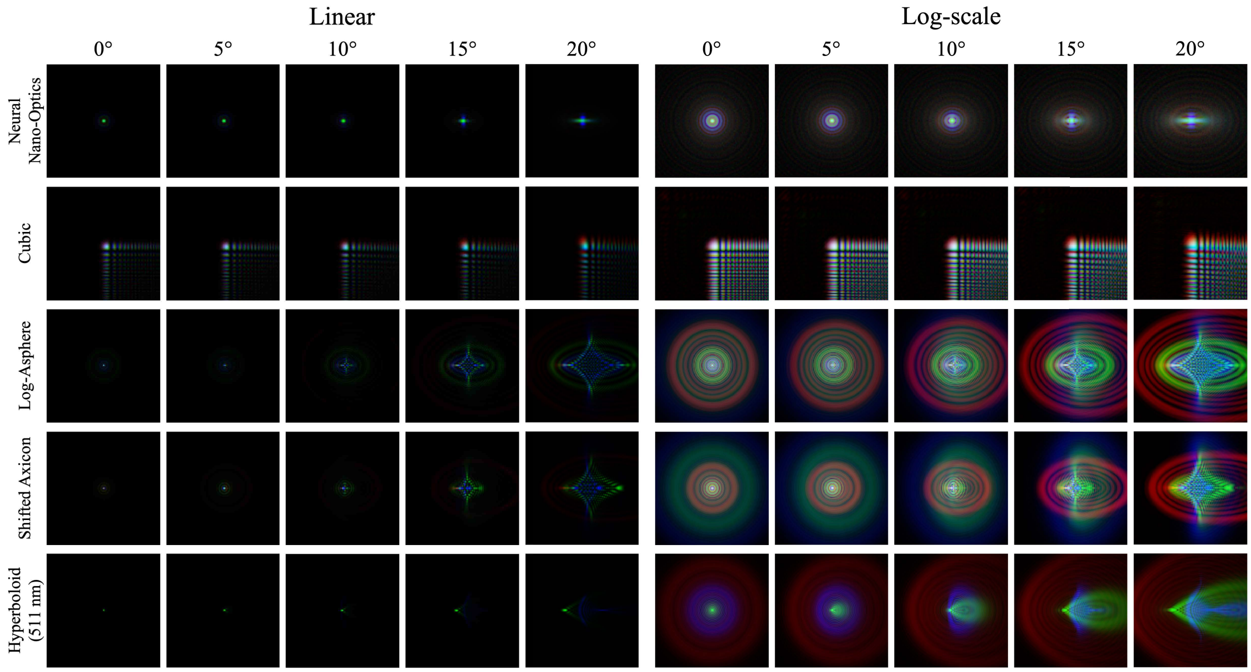

Supplementary Figure 11: Meta-optic PSFs. In this figure we show the simulated PSFs for each design. Our optimization produces a meta-optic whose spatial PSFs are compact across all wavelengths and are slowly varying across field angles. Other designs exhibit PSFs with greater blur, especially for the red wavelength, and with rapid variation across field angles. The PSF corresponding to the cubic design contains large tails that cause the overall size of the PSF to be much larger than that of all other designs. As such, we only display the center crop of cubic design PSFs from Colburn et al.[3]

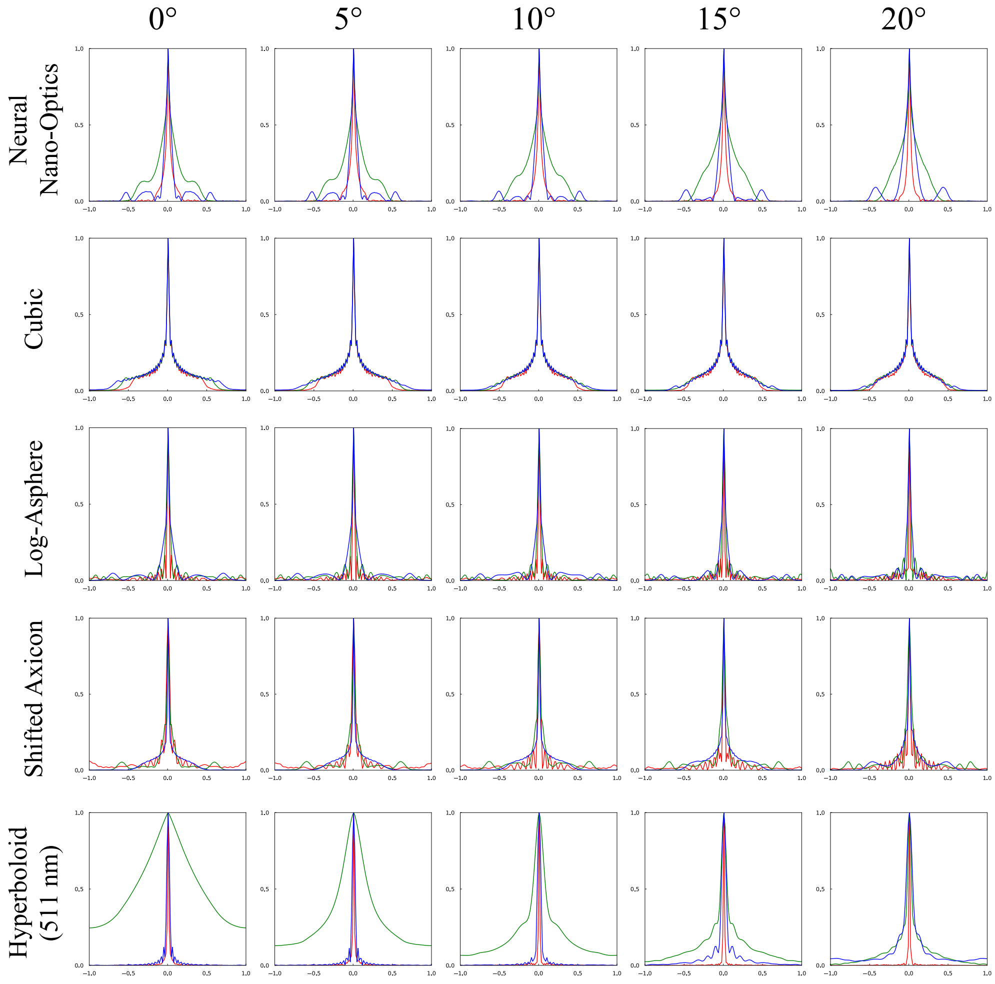

Supplementary Figure 12: Meta-optic MTFs. In this figure we show the simulated MTFs for each design at field angles 0°, 5°, 10°, 15°, 20°.

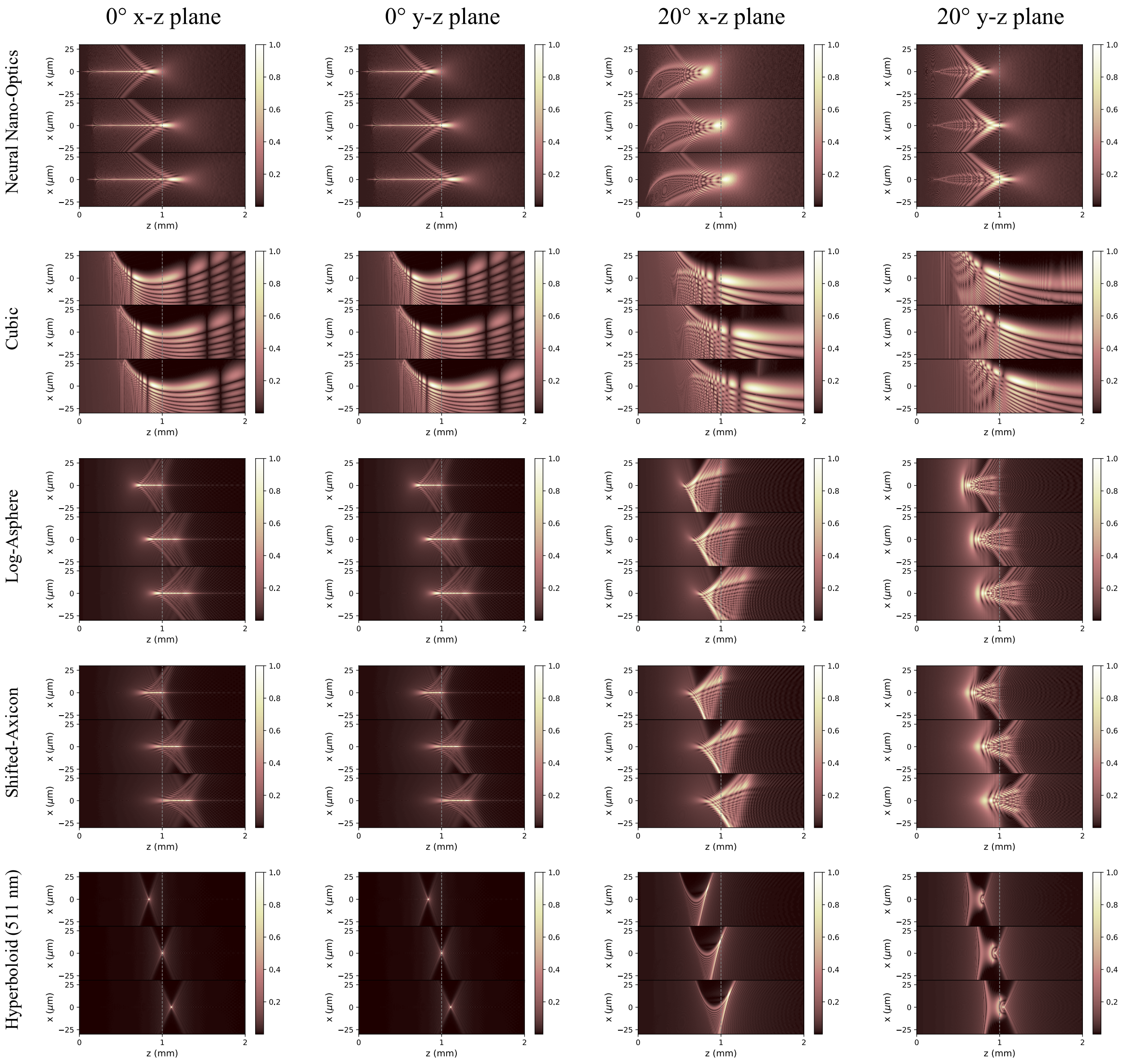

Supplementary Figure 13: Meta-optic Intensity Profiles. In this figure we show intensity profiles in the y-z and x-z planes for normal incidence and at 20°. The optical intensity for 462 nm, 511 nm, 606 nm are shown from top to bottom for each plot. Our end-to-end optimized meta-optic exhibits stable performance across all wavelengths and field angles while avoiding severe aberrations incurred by the cubic design from Colburn et al.[3]

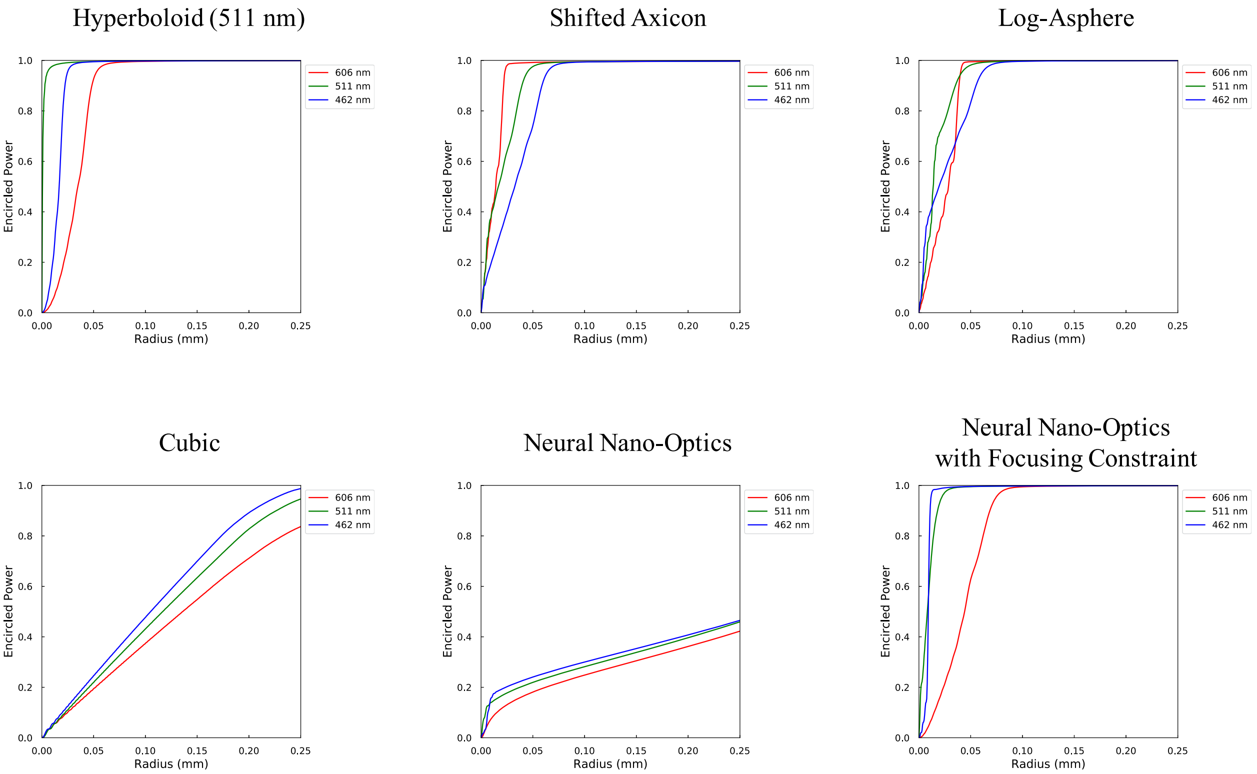

Supplementary Figure 14: Meta-optic Simulated Diffraction Efficiencies. In this figure we show diffraction efficiencies for the meta-optics within the visible regime. The efficiency here is defined as the ratio of power enclosed within a circle of a specified radius (x-axis in the plot) at the focal plane to the power incident on the meta-optic. We observe that the learned design actually exhibits slightly reduced focusing efficiency, demonstrating that focusing efficiency is not a necessary merit for high-quality imaging. Nevertheless, we can boost the efficiency by incorporating additional constraints in our optimization routine to maximize the enclosed power within the PSF. This is shown in the plot titled "Neural Nano-Optics with Focusing Constraint."

Supplementary Note 9: Validation of Neural Feature Propagation

Existing Deconvolution Algorithms We compare our deconvolution method against existing deconvolution algorithms, including traditional deconvolution methods and learning based approaches. As traditional deconvolution methods, we compare against Wiener filtering[26], Richardson-Lucy[27], and Alternating Direction Method of Multipliers (ADMM) optimization with an $\mathcal{L}_1$ total variation prior[14]. For the learning based approaches, we compare against the non-blind deconvolution approach from Son et al.[28] and the blind deconvolution approach from Kupyn et al.[16]. Our neural feature propagation achieves a fast runtime of 58 ms per 720×720 RGB image that allows for real-time applications.

Deconvolution Algorithm Assessment We perform synthetic experiments to evaluate the efficacy of the deconvolution algorithms. For evaluation, we utilize our proposed learned meta-optic in addition to the aforementioned cubic and log-asphere designs. We finetune the deconvolution algorithms to each of these designs and we evaluate using the same offset PSF scheme as described earlier in the meta-optics evaluation. Quantitative results are shown in Supplementary Table 11 and qualitative results are shown in Supplementary Figs. 15 and 16. Our neural feature propagation deconvolution achieves the highest quantitative metrics across all evaluation settings, validating its effectiveness. This method even performs well for meta-optic designs that were not obtained with our end-to-end optimization scheme, as can be seen when we apply it to the cubic and log-asphere designs, which demonstrates the generalizability of our algorithm.

The finetuning procedure for each deconvolution method is performed as follows. For Son et al.[28] and Kupyn et al.[16], we train their deconvolution networks on the synthetically blurred sensor images using the same hyperparameter settings as described in their work. For ADMM deconvolution we manually tune the hyperparameters to achieve the best results. We set the gradient sparsity prior's coefficient to be 4×10^{-6}, the initial consensus term to be 1×10^{-6}, the ratio parameter for updating the consensus term to be 2.7, and the maximum number of iterations at 10. For Richardson-Lucy we set the maximum number of iterations at 10. For Wiener filtering, we set the SNR value at 1×10^{4}.

We observed that traditional deconvolution algorithms are not robust to diverse aberrations commonly encountered in meta-optics imaging. On the other hand, the blind deep-learning method of Kupyn et al.[16] falls into a local minima where it does not do any deblurring at all. The method of Son et al.[28] performs better as it combines traditional deconvolution algorithms with deep learning approaches. However, they do not employ a feature extractor and only apply deep learning layers after an initial Wiener filtering step. As such, they are unable to extract and utilize salient information embedded within the sensor measurement as well as our neural feature propagation method.

Supplementary Table 11: Quantitative comparison between different deconvolution algorithms in simulation. We apply the deconvolution algorithms to different meta-optic designs and with varying PSF field angles. Our neural feature propagation algorithm achieves the highest reconstruction quality across all metrics and meta-optic designs, even for designs that were not obtained with our end-to-end optimization pipeline. This is validated by how we outperform all other deconvolution methods by $4\,\text{dB}$ PSNR and with $2.5\times$ lower MSE on the log-asphere deconvolution task (bottom row).

Meta-Optics Design	Image Formation PSF Field Angle θ_{conv}	Deconvolution PSF Field Angle θ_{deconv}	Algorithm	MSE	PSNR (dB)	SSIM
Neural Nano-Optics	5°	0°	Neural Feature Propagation	**0.00185**	**27.7**	**0.839**
			Son et al.[28]	0.00345	24.8	0.792
			Kupyn et al.[16]	0.01398	19.0	0.575
			ADMM with $\mathcal{L}_1$-TV[14]	0.00329	25.9	0.709
			Richardson-Lucy[27]	0.00550	23.5	0.643
			Wiener filtering[26]	0.00669	21.8	0.336
Neural Nano-Optics	15°	10°	Neural Feature Propagation	**0.00273**	**26.1**	**0.793**
			Son et al.[28]	0.00510	23.2	0.723
			Kupyn et al.[16]	0.01498	18.7	0.565
			ADMM with $\mathcal{L}_1$-TV[14]	0.00465	24.3	0.661
			Richardson-Lucy[27]	0.00629	22.8	0.630
			Wiener filtering[26]	0.01414	18.8	0.243
Cubic[3]	0°	0°	Neural Feature Propagation	**0.00334**	**25.1**	**0.740**
			Son et al.[28]	0.00618	22.3	0.661
			Kupyn et al.[16]	0.02693	16.0	0.517
			ADMM with $\mathcal{L}_1$-TV[14]	0.00478	23.9	0.659
			Richardson-Lucy[27]	0.01252	19.5	0.557
			Wiener filtering[26]	0.01653	17.8	0.188
Log-Asphere[24]	5°	0°	Neural Feature Propagation	**0.00217**	**27.3**	**0.811**
			Son et al.[28]	0.00435	24.1	0.707
			Kupyn et al.[16]	0.01332	19.4	0.556
			ADMM with $\mathcal{L}_1$-TV[14]	0.00605	22.9	0.628
			Richardson-Lucy[27]	0.00858	21.5	0.583
			Wiener filtering[26]	0.02634	15.8	0.128
Log-Asphere[24]	15°	10°	Neural Feature Propagation	**0.00395**	**24.7**	**0.748**
			Son et al.[28]	0.01132	20.1	0.529
			Kupyn et al.[16]	0.01612	18.5	0.535
			ADMM with $\mathcal{L}_1$-TV[14]	0.02001	17.4	0.484
			Richardson-Lucy[27]	0.01248	19.7	0.545
			Wiener filtering[26]	0.04202	13.9	0.066

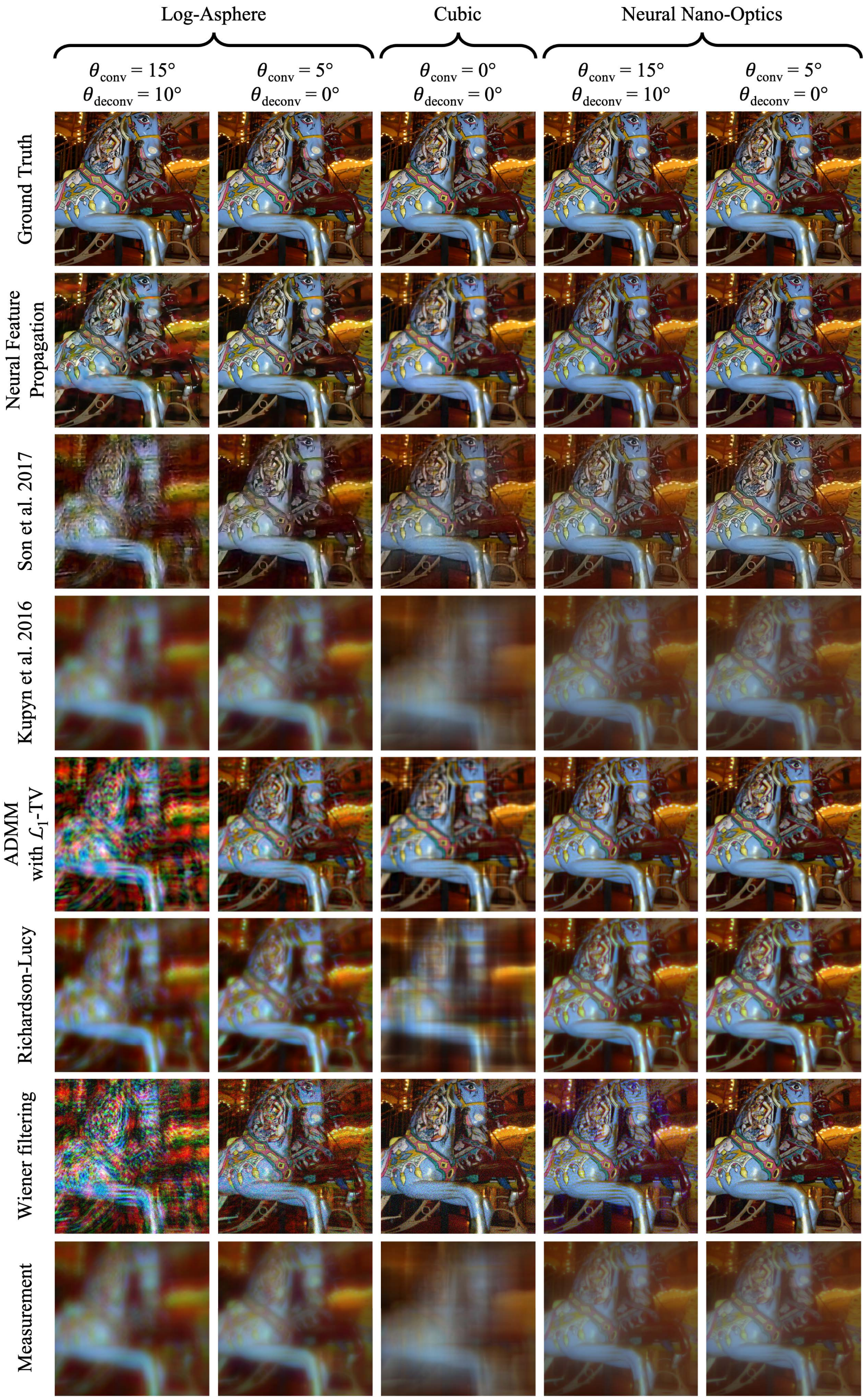

Supplementary Figure 15: Qualitative comparison between deconvolution algorithms in simulation. Wiener filtering exacerbates sensor noise. Richardson-Lucy and ADMM both fail to recover sharp fine details. Kupyn et al.[16] falls into a local minima where it avoids doing any deblurring. Son et al.[28] do not employ a multi-scale feature extractor and consequently they do not reach the same level of performance as our method. Neural feature propagation recovers high frequency content and reproduces accurate color while mitigating sensor noise.

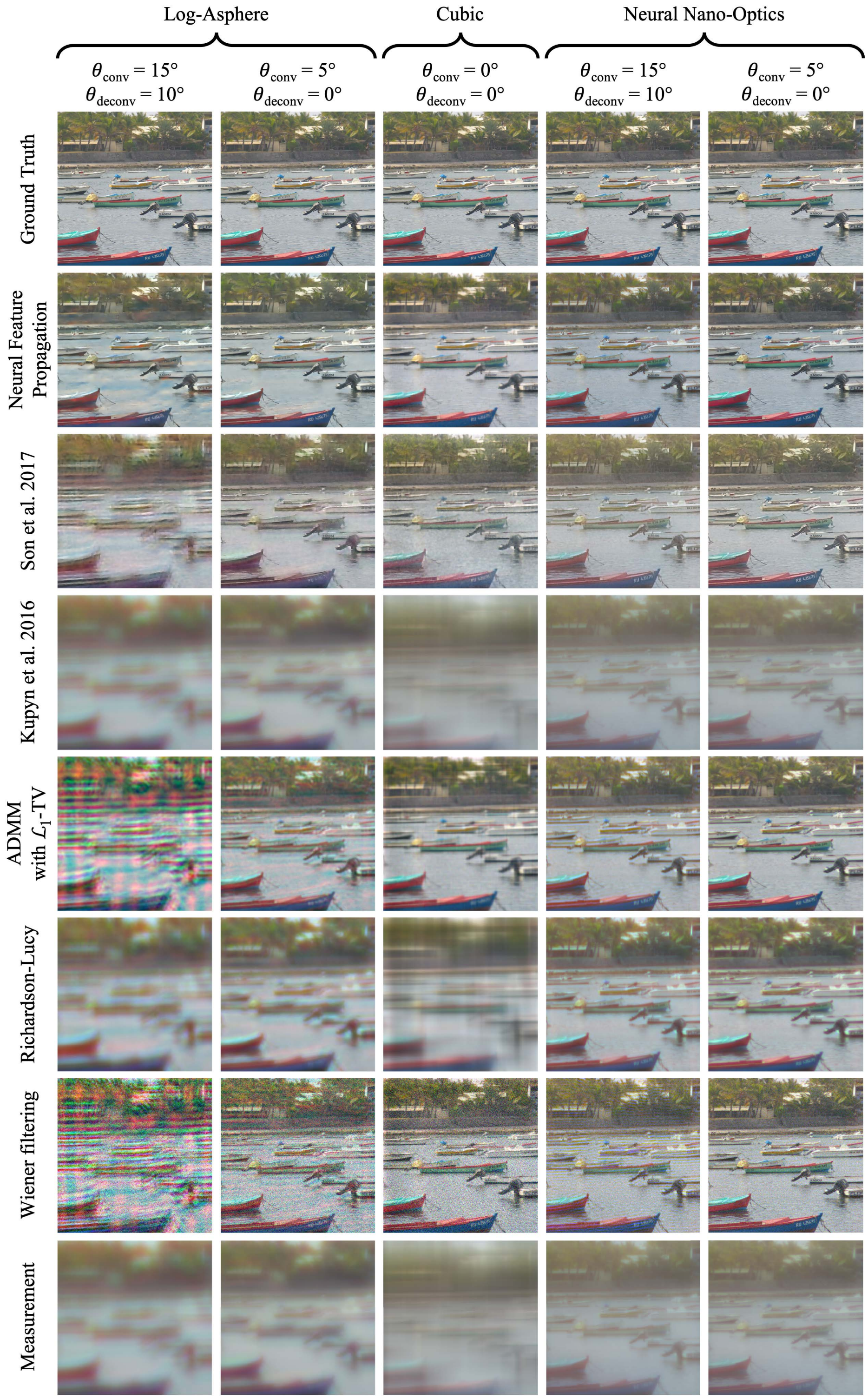

Supplementary Figure 16: Qualitative comparison between deconvolution algorithms in simulation. Wiener filtering exacerbates sensor noise. Richardson-Lucy and ADMM both fail to recover sharp fine details. Kupyn et al.[16] falls into a local minima where it avoids doing any deblurring. Son et al.[28] do not employ a multi-scale feature extractor and consequently they do not reach the same level of performance as our method. Neural feature propagation recovers high frequency content and reproduces accurate color while mitigating sensor noise.

Supplementary Note 10: Spatial Resolution Evaluation

We evaluate the spatial resolution of our meta-optic imager using a Siemens Star chart and a USAF 1951 chart, results are shown in Supplementary Figs. 17 and 18. As was done for the imaging experiments, our display to optic distance is 120 mm. Our neural nano-optic imager is able to resolve line pairs up to Group 0, Element 6 of the USAF 1951 chart for all color channels, thus providing us with a spatial resolution of 213.6 lp/mm. While the hyperboloid meta-optic allows for high spatial resolution in the green wavelength, the other channels are aberrated such that none of the line pairs in the USAF 1951 chart can be read. Similarly for Colburn et al.[3], severe chromatic aberrations prevent resolution of line pairs in the USAF 1951 chart, thus limiting the resolution to Group -2, Element 1 which corresponds to 30 lp/mm.

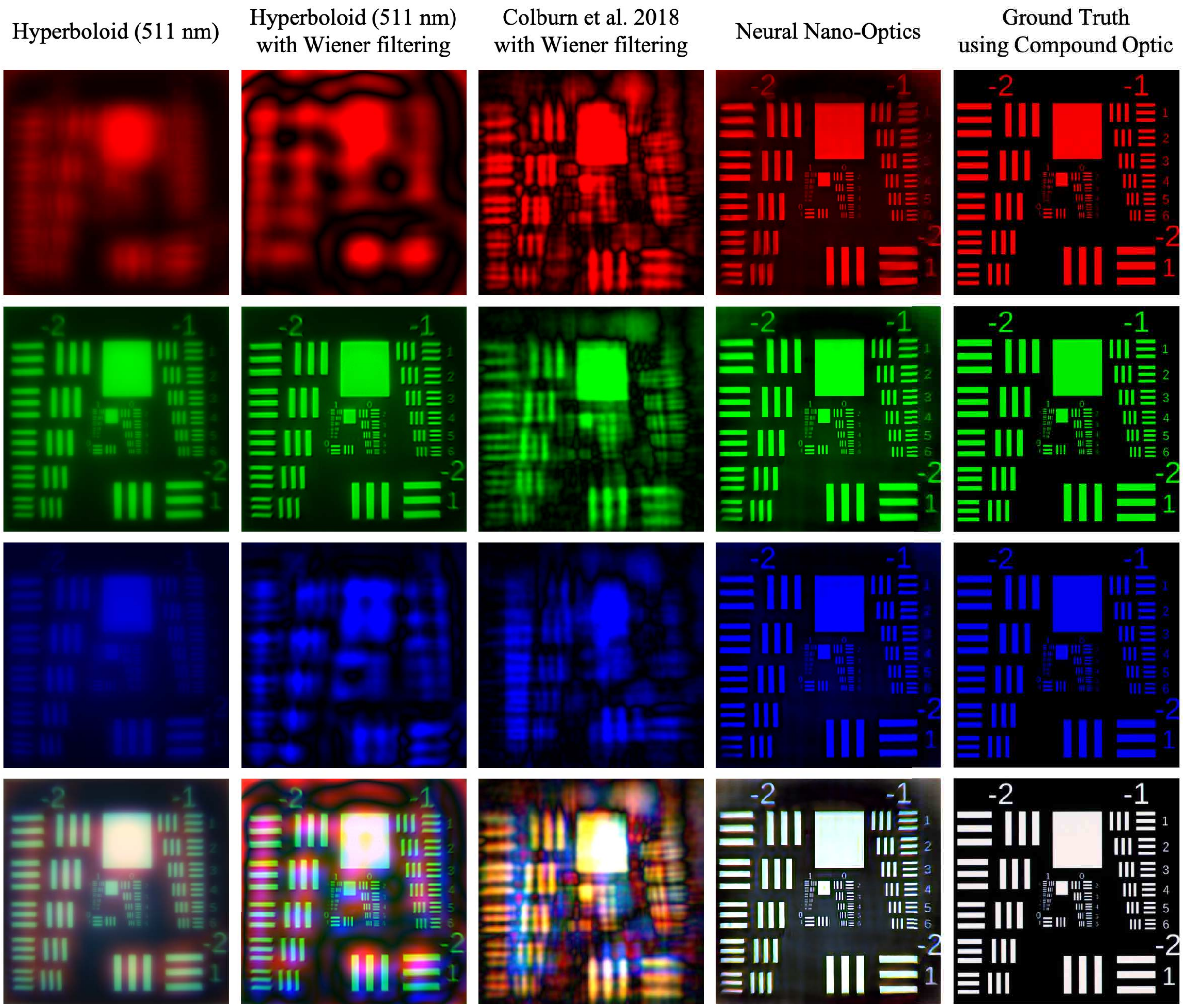

Supplementary Figure 17: Qualitative results for reconstructions of the USAF 1951 chart. The reconstruction with our neural nano-optic imager accurately is able to resolve line pairs up to Group 0, Element 6 across all color channels whereas the other meta-optic imagers suffer from aberrations outside of the nominal wavelength range.

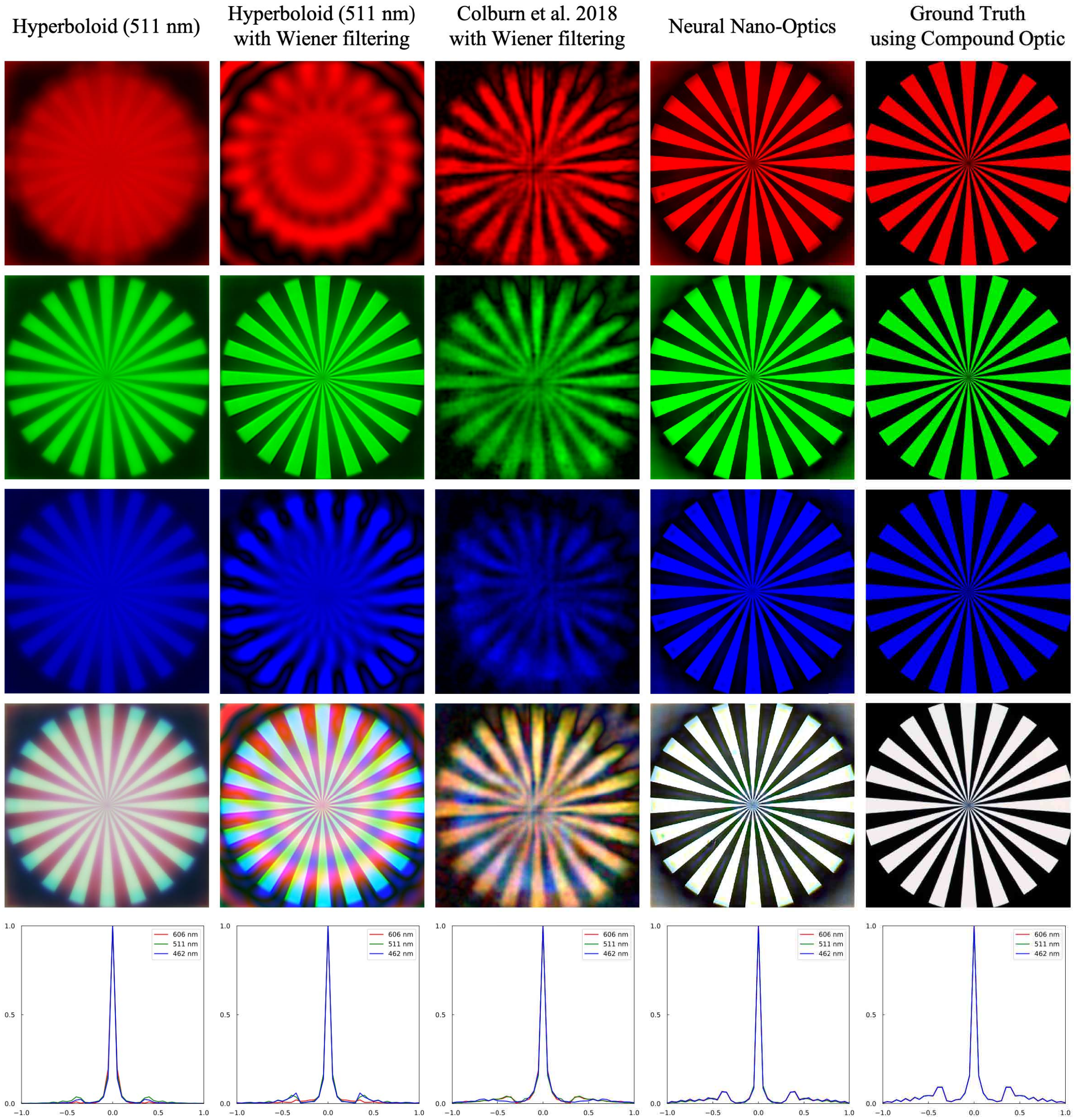

Supplementary Figure 18: Qualitative results for reconstructions of the Siemens Star chart with corresponding MTF plots. The reconstruction with our neural nano-optic imager accurately matches the MTF of the ground truth Siemens Star whereas the MTFs of the other methods lose significant spatial resolution.

Supplementary Note 11: Additional Experimental Results

Our image reconstructions using neural nano-optics achieves 22.1 dB PSNR and 0.807 SSIM on full-color reconstructions with respect with to a ground truth acquired with a high-quality compound optic. Quantitative metrics and comparisons are shown in Supplementary Table 12. We achieve $10\times$ lower reconstruction error, measured by pixel-wise mean squared error (MSE), and improve by over 10 dB PSNR on the red channel reconstruction, which is an order of magnitude improvement over meta-optics that are designed for a specific wavelength (usually the green channel). This improvement holds even over Colburn et al.[3] which was designed to maintain consistent performance across the visible spectrum. We also evaluate using recent learned perceptual metrics, specifically the Learned Perceptual Image Patch Similarity (LPIPS) metric[20]. These metrics compute differences between extracted features of images as opposed to differences between the image intensities themselves. We again demonstrate lower reconstruction error with this metric. Additional qualitative experimental reconstruction results are shown in Supplementary Figs. 19, 20, 21, 22, 23, and corresponding sensor measurements are shown in Supplementary Figs. 24 and 25. We showcase comparisons against images acquired using a hyperboloid meta-optic designed for 511 nm with Wiener filtering, Colburn et al.[3] with Wiener filtering, the neural nano-optic using the proposed feature propagation deconvolution, and a highly corrected six-element compound optical stack (Edmund Optics 50mm C Series #59-873). We additionally show comparisons against a conventional refractive lens (Thorlabs LA1540) and demonstrate that our nano-optic imager achieves comparable performance despite being $10000\times$ smaller. Lastly, we also show comparisons against a 500 µm aperture version of the cubic meta-optic from Colburn et al.[3] Although the larger aperture does improve image quality, the artifacts induced by the cubic design are still present.

We observe substantial image quality improvement across a diverse range of scenes. In the first row of Supplementary Fig. 19, we observe faithful reconstruction of a colored buckets and the third row shows a clear reconstruction of the hotel, including cloud details in the sky. The middle row of Supplementary Fig. 20 shows clear reconstruction of a highly detailed toy which the other meta-optic methods struggle to recover. Detailed urban scenes are recovered in the middle row of Supplementary Fig. 21 and the first row of Supplementary Fig. 22. We see that recovered text details are legible in the first row of Supplementary Fig. 21 and the middle row of Supplementary Fig. 22.

Supplementary Table 12: Quantitative evaluation of *experimental captures and reconstructions*. Evaluation metrics are computed with respect to the image acquired with the compound optic. Note that lower is better for MSE and LPIPS. Wiener filtering with manually optimized SNR parameters is applied to Colburn et al.[3] and to the traditional meta-optic for 511 nm. We also compare against a conventional refractive lens and we achieve similar quantitative performance which further validates the imaging capability of our neural nano-optics. The compound optic, conventional refractive lens, and Colburn et al.[3] are resized to the resolution of the 500 µm aperture metasurface reconstructions.

	Red (606 nm)		Green (511 nm)		Blue (462 nm)		Full-Color			
Method	MSE	PSNR	MSE	PSNR	MSE	PSNR	MSE	PSNR	SSIM	LPIPS[20]
**Neural Nano-Optics**	**0.00479**	**23.9**	**0.00621**	**23.5**	**0.00978**	**20.5**	**0.00693**	**22.1**	**0.807**	**0.2082**
Colburn et al.[3] with Wiener filtering	0.04987	13.2	0.03352	15.1	0.04943	13.5	0.04427	13.7	0.625	0.3450
Traditional Meta-Optic with Wiener filtering	0.04944	13.8	0.01430	19.3	0.03233	16.2	0.03203	15.8	0.676	0.2805
Traditional Meta-Optic	0.05308	13.7	0.02773	16.4	0.02910	16.5	0.03664	15.2	0.688	0.2635
Conventional Refractive Lens	0.00426	24.2	0.00494	24.0	0.00952	21.7	0.00624	23.1	0.800	0.1945

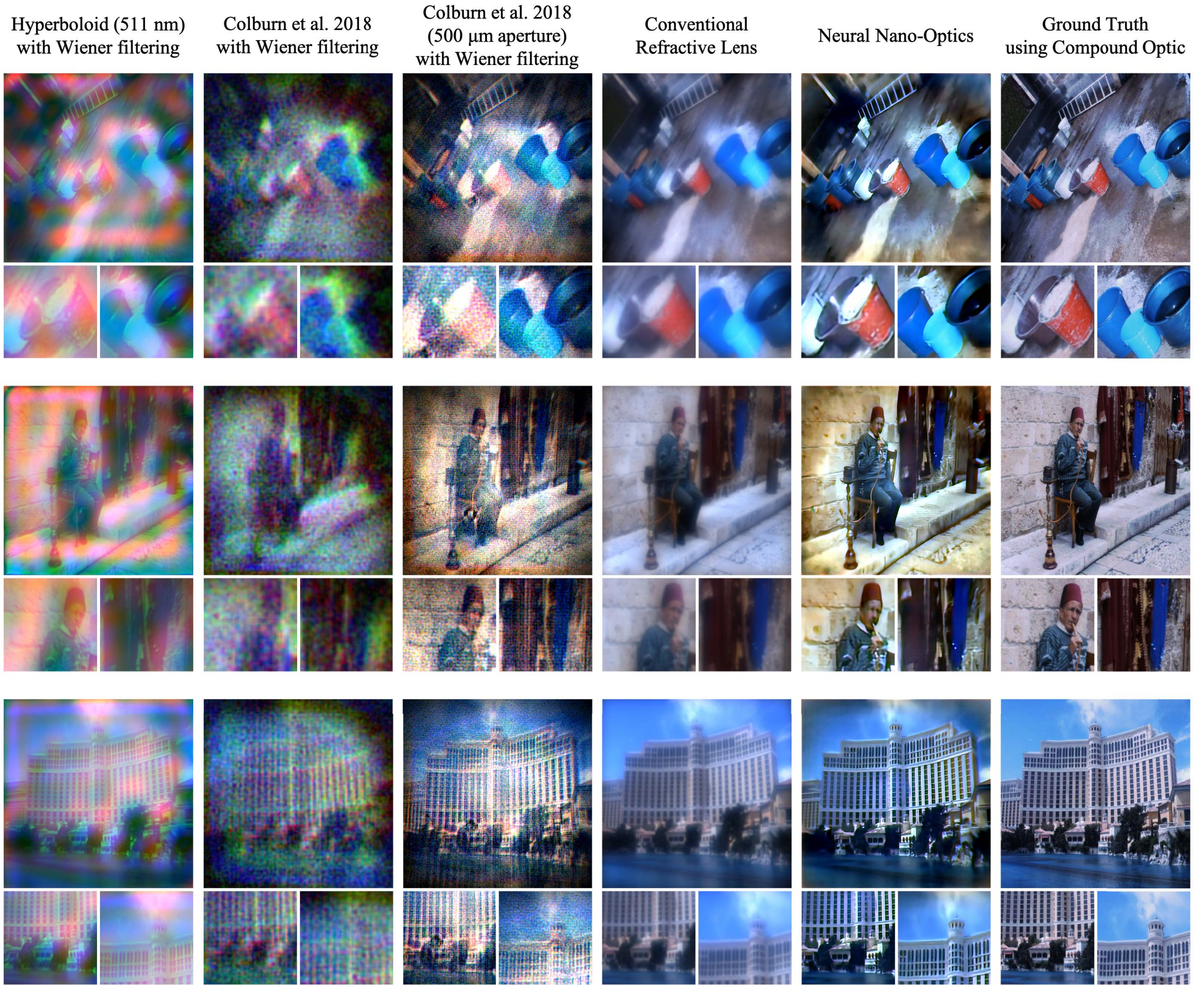

Supplementary Figure 19: Additional experimental reconstruction results. These results supplement the reconstruction results from Fig. 2 of the main manuscript.

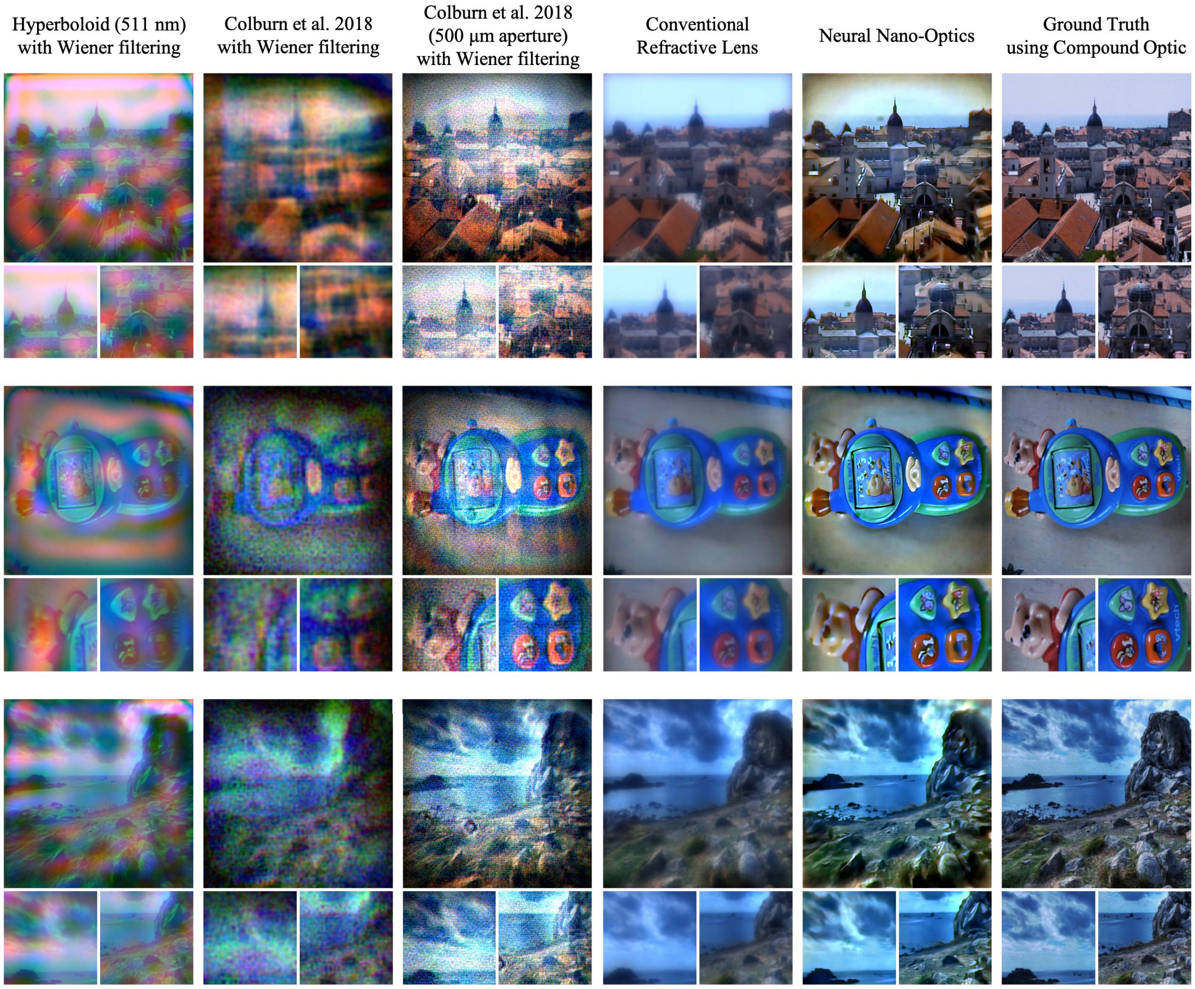

Supplementary Figure 20: Additional experimental reconstruction results. These results supplement the reconstruction results from Fig. 2 of the main manuscript.

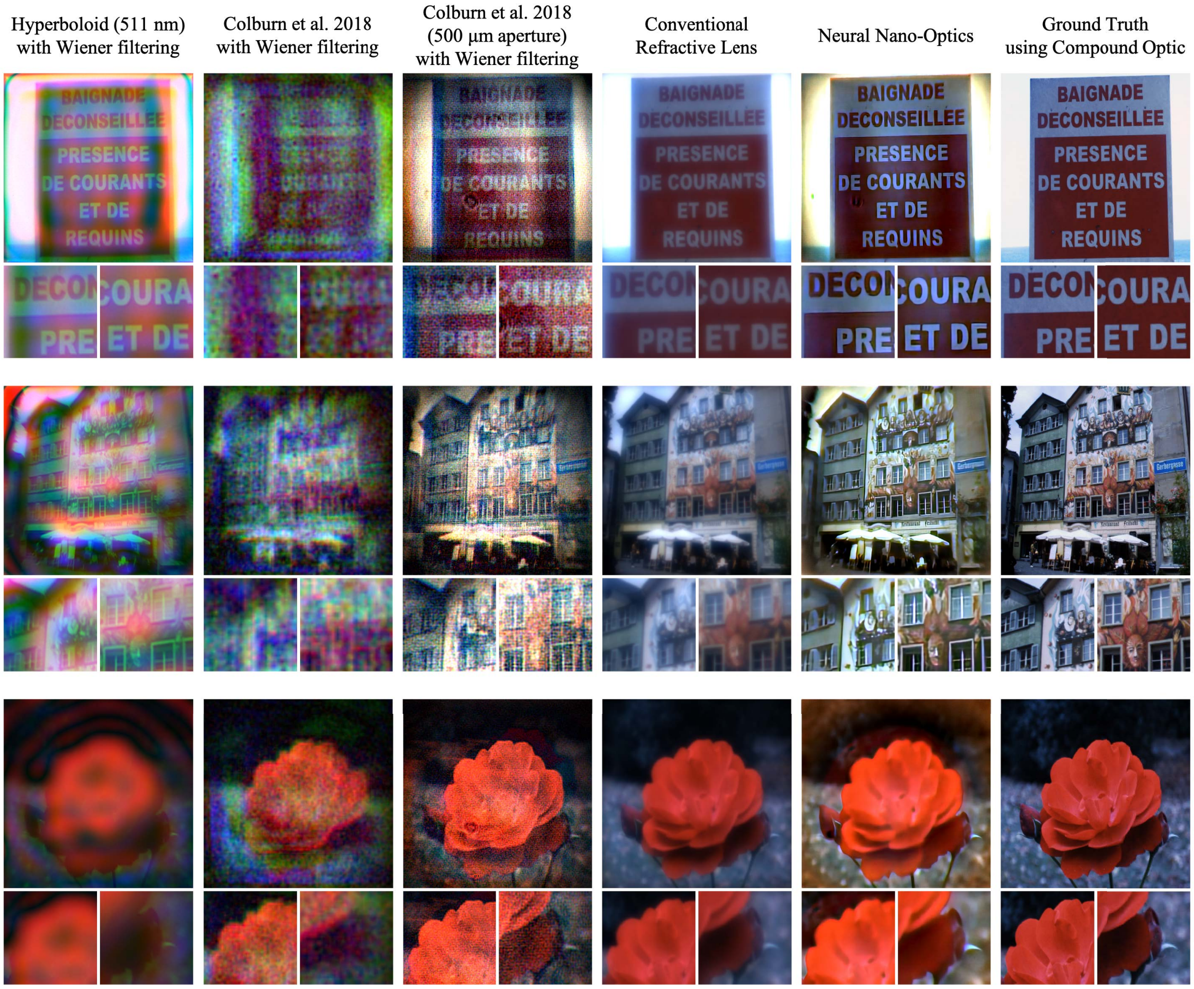

Supplementary Figure 21: Additional experimental reconstruction results. These results supplement the reconstruction results from Fig. 2 of the main manuscript.

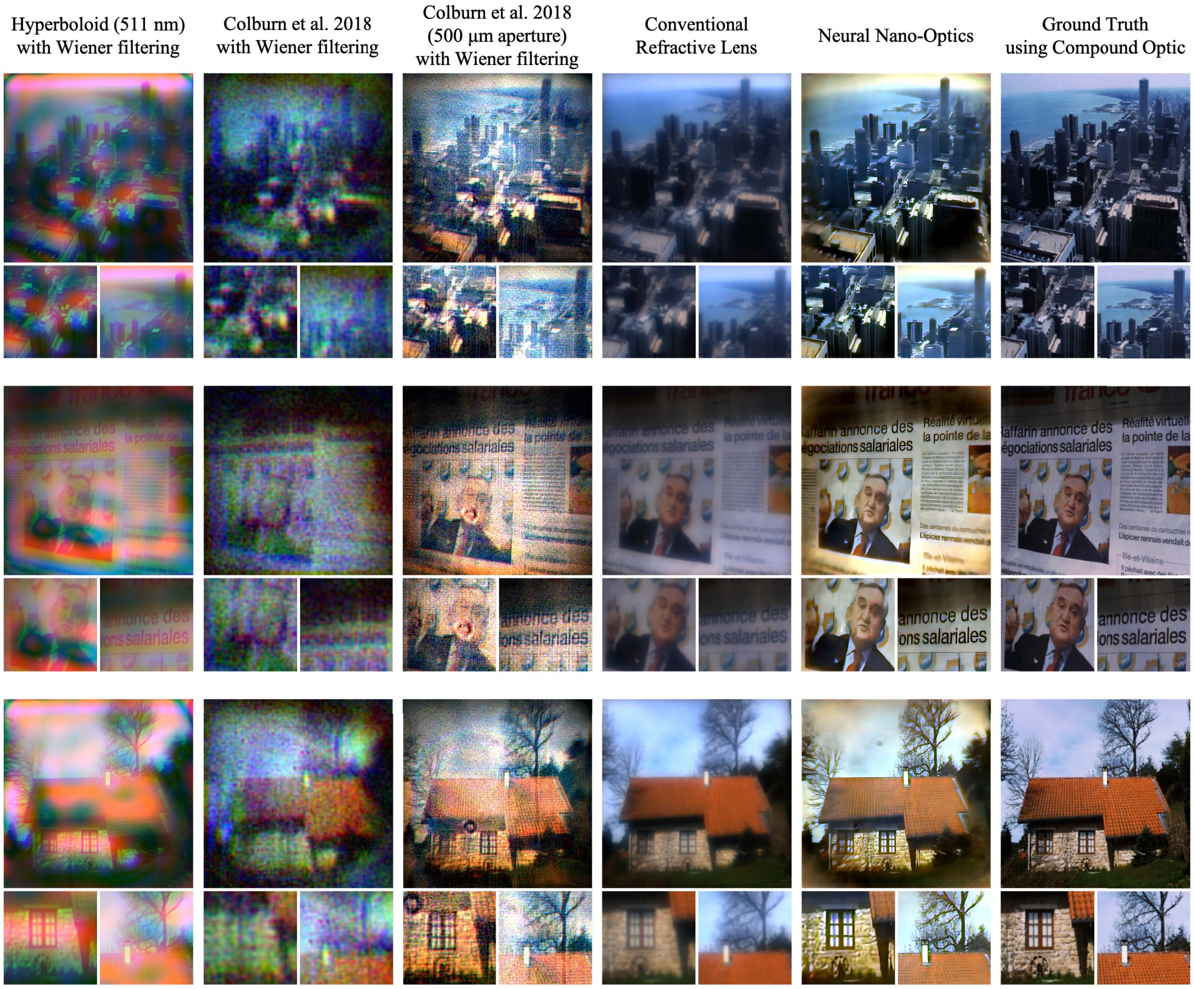

Supplementary Figure 22: Additional experimental reconstruction results. These results supplement the reconstruction results from Fig. 2 of the main manuscript.

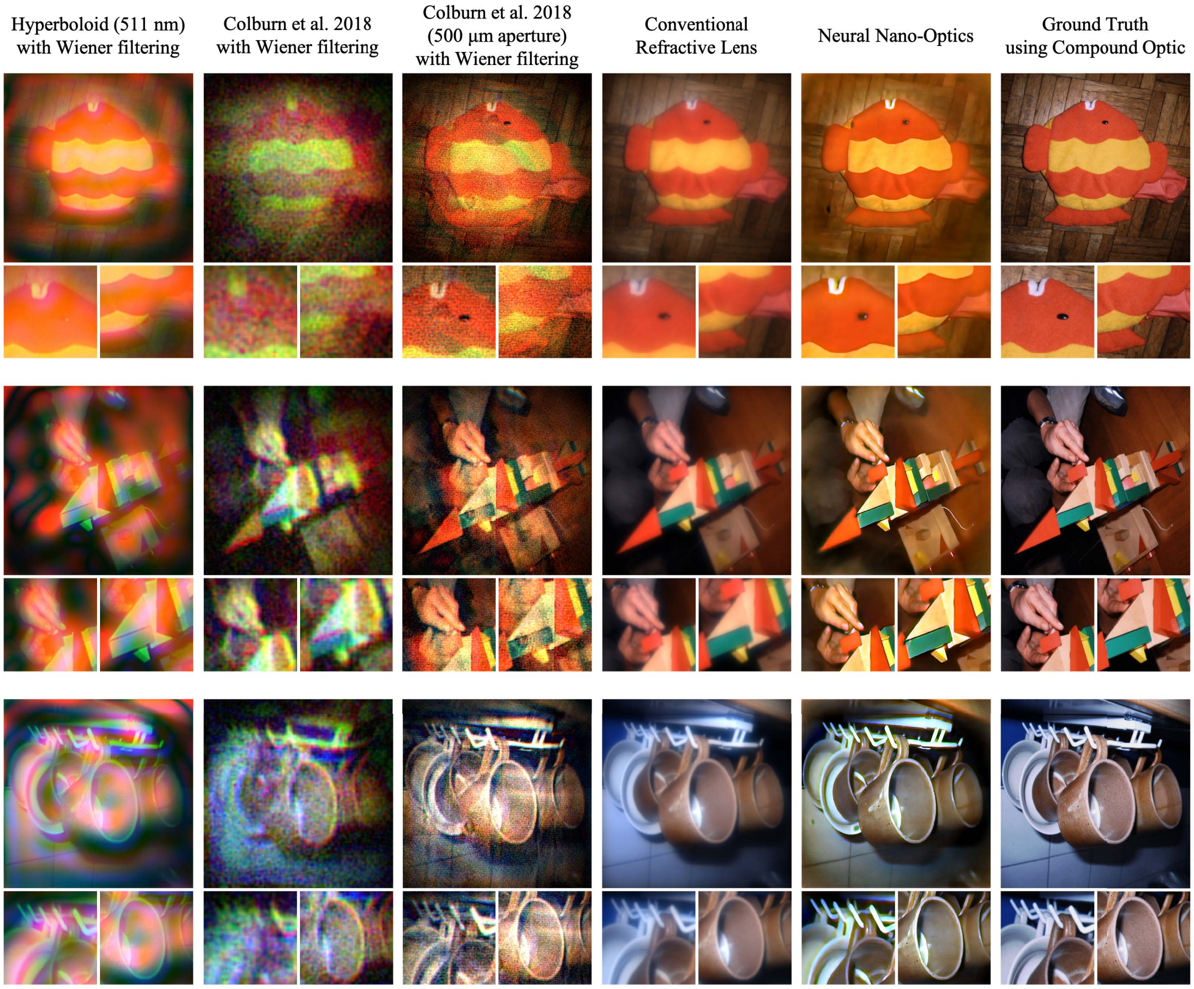

Supplementary Figure 23: Additional experimental reconstruction results. These results supplement the reconstruction results from Fig. 2 of the main manuscript.

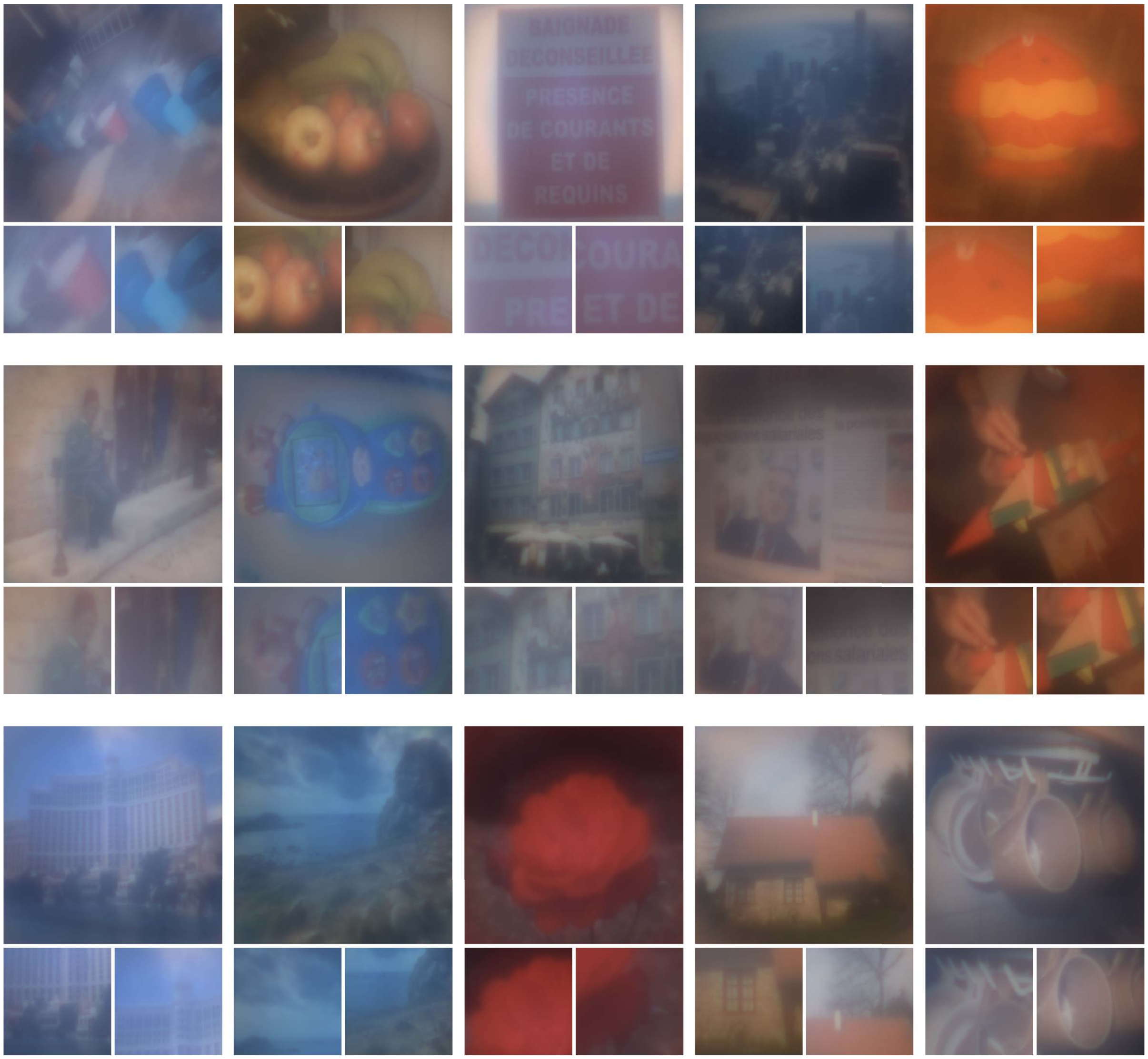

Supplementary Figure 24: Experimental measurements for our neural nano-optics corresponding to the reconstructions shown in Supplementary Figs. 19, 20, 21, 22, 23.

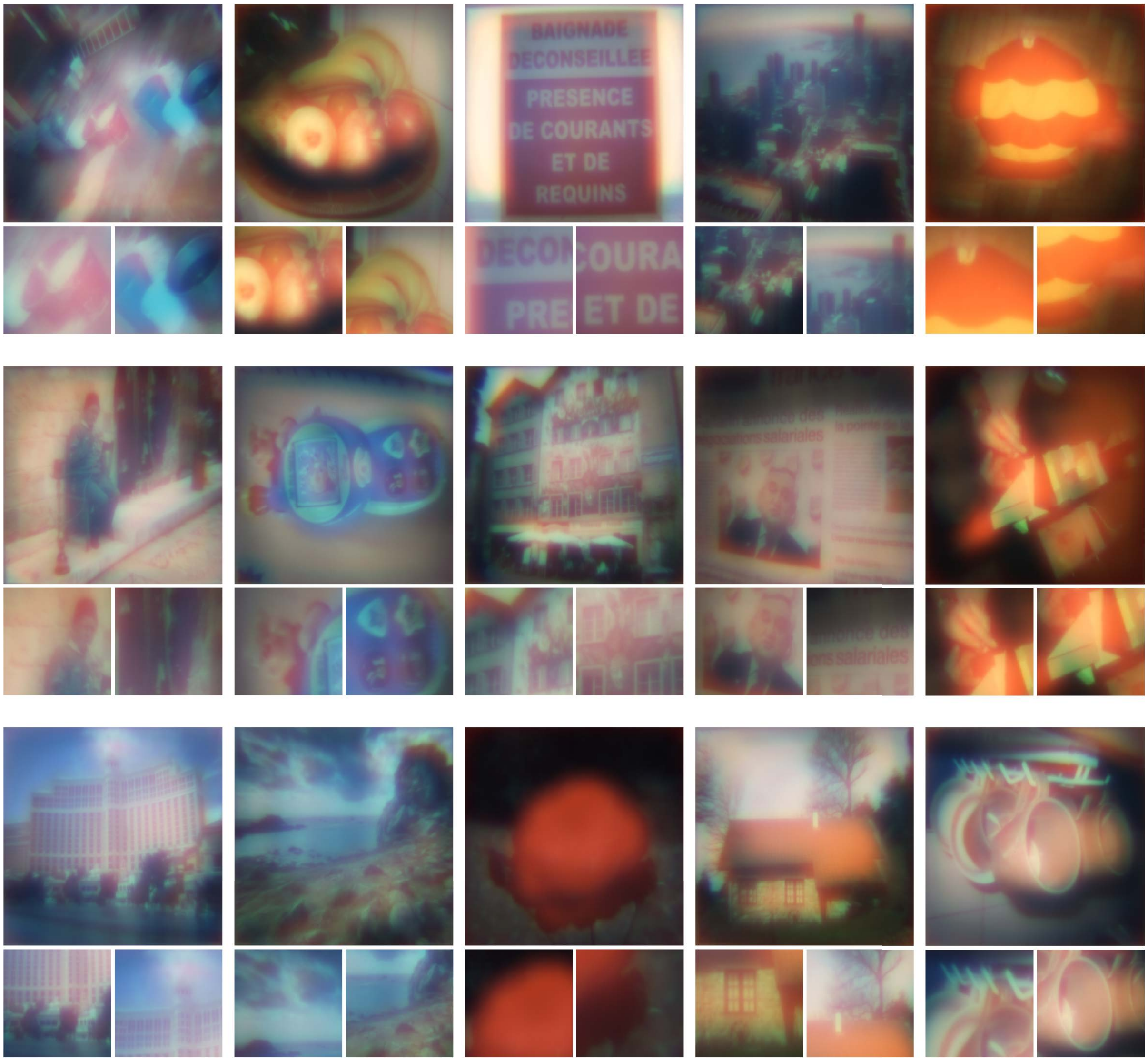

Supplementary Figure 25: Experimental measurements for the traditional meta-optic designed for 511 nm corresponding to the reconstructions shown in Supplementary Figs. 19, 20, 21, 22, 23.

Supplementary References

1. Ndao, A. *et al.* Octave bandwidth photonic fishnet-achromatic-metalens. *Nature Communications* **11**, 3205 (2020).

2. Chen, W. T., Zhu, A. Y., Sisler, J., Bharwani, Z. & Capasso, F. A broadband achromatic polarization-insensitive metalens consisting of anisotropic nanostructures. *Nature Communications* **10**, 355 (2019).

3. Colburn, S., Zhan, A. & Majumdar, A. Metasurface optics for full-color computational imaging. *Science Advances* **4**, eaar2114 (2018).

4. Chen, W. *et al.* A broadband achromatic metalens for focusing and imaging in the visible. *Nature Nanotechnology* **13**, 220–226 (2018).

5. Wang, S. *et al.* A broadband achromatic metalens in the visible. *Nature Nanotechnology* **13**, 227–232 (2018).

6. Shrestha, S., Overvig, A. C., Lu, M. Y., Stein, A. & Yu, N. Broadband achromatic dielectric metalenses. *Light: Science & Applications* **7**, 85 (2018).

7. Khorasaninejad, M. *et al.* Achromatic metalens over 60 nm bandwidth in the visible and metalens with reverse chromatic dispersion. *Nano Letters* **17**, 1819–1824 (2017).

8. Wang, S. *et al.* Broadband achromatic optical metasurface devices. *Nature Communications* **8**, 187 (2017).

9. Arbabi, E., Arbabi, A., Kamali, S. M., Horie, Y. & Faraon, A. Controlling the sign of chromatic dispersion in diffractive optics with dielectric metasurfaces. *Optica* **4**, 625–632 (2017).

10. Foi, A., Trimeche, M., Katkovnik, V. & Egiazarian, K. Practical poissonian-gaussian noise modeling and fitting for single-image raw-data. *IEEE Transactions on Image Processing* **17**, 1737–1754 (2008).

11. Kingma, D. P. & Welling, M. Auto-encoding variational bayes. In *Proc. International Conference on Learning Representations (ICLR)* (2014).

12. Williams, R. J. Simple statistical gradient-following algorithms for connectionist reinforcement learning. *Machine Learning* **8**, 229–256 (1992).

13. Krishnan, D. & Fergus, R. Fast image deconvolution using hyper-laplacian priors. In *Proc. Advances in Neural Information Processing Systems* (2009).

14. Bioucas-Dias, J. M., Figueiredo, M. A. & Oliveira, J. P. Total variation-based image deconvolution: a majorization-minimization approach. In *Proc. IEEE International Conference on Acoustics Speech and Signal Processing (ICASSP)* (2006).

15. Boyd, S., Parikh, N. & Chu, E. *Distributed optimization and statistical learning via the alternating direction method of multipliers* (2011).

16. Kupyn, O., Budzan, V., Mykhailych, M., Mishkin, D. & Matas, J. Deblurgan: Blind motion deblurring using conditional adversarial networks. In *Proc. IEEE Conference on Computer Vision and Pattern Recognition (CVPR)* (2018).

17. Reeves, S. Fast image restoration without boundary artifacts. *IEEE Transactions on Image Processing* **14**, 1448–1453 (2005).

18. He, K., Zhang, X., Ren, S. & Sun, J. Deep residual learning for image recognition. In *Proc. IEEE Conference on Computer Vision and Pattern Recognition (CVPR)* (2016).

19. Johnson, J., Alahi, A. & Fei-Fei, L. Perceptual losses for real-time style transfer and super-resolution. In *Proc. European Conference on Computer Vision (ECCV)* (2016).

20. Zhang, R., Isola, P., Efros, A. A., Shechtman, E. & Wang, O. The unreasonable effectiveness of deep features as a perceptual metric. In *Proc. IEEE International Conference on Computer Vision and Pattern Recognition (CVPR)* (2018).

21. Wang, Z., Bovik, A., Sheikh, H. R. & Simoncelli, E. P. Image quality assessment: from error visibility to structural similarity. *IEEE Transactions on Image Processing* **13**, 600–612 (2004).

22. Kingma, D. & Ba, J. Adam: A method for stochastic optimization. In *Proc. International Conference on Learning Representations (ICLR)* (2015).

23. Jégou, H., Douze, M. & Schmid, C. Hamming embedding and weak geometric consistency for large scale image search. In *Proc. European Conference on Computer Vision (ECCV)* (2008).

24. Chi, W. & George, N. Electronic imaging using a logarithmic asphere. *Optics Letters* **26**, 875–877 (2001).

25. Wang, Y., Yan, S., Friberg, A. T., Kübel, D. & Visser, T. D. Electromagnetic diffraction theory of refractive axicon lenses. *Journal of the Optical Society of America* **34**, 1201–1211 (2017).

26. Wiener, N. Extrapolation, interpolation, and smoothing of stationary time series (1964).

27. Richardson, W. H. Bayesian-based iterative method of image restoration. *Journal of the Optical Society of America* **62**, 55–59 (1972).

28. Son, H. & Lee, S. Fast non-blind deconvolution via regularized residual networks with long/short skip-connections. In *Proc. IEEE International Conference on Computational Photography (ICCP)* (2017).
